# Complex Interplay of Evolutionary Forces in the *ladybird* Homeobox Genes of *Drosophila melanogaster*

**DOI:** 10.1371/journal.pone.0022613

**Published:** 2011-07-22

**Authors:** Evgeniy S. Balakirev, Maria Anisimova, Francisco J. Ayala

**Affiliations:** 1 Department of Ecology and Evolutionary Biology, University of California Irvine, Irvine, California, United States of America; 2 A.V. Zhirmunsky Institute of Marine Biology, Far Easter Branch, Russian Academy of Sciences, Vladivostok, Russia; 3 Department of Computer Science, Swiss Federal Institute of Technology ETH Zürich, Zürich, Switzerland; 4 Swiss Institute of Bioinformatics, Lausanne, Switzerland; University of Arkanas, United States of America

## Abstract

Tandemly arranged paralogous genes *lbe* and *lbl* are members of the *Drosophila* NK homeobox family. We analyzed population samples of *Drosophila melanogaster* from Africa, Europe, North and South America, and single strains of *D. sechellia*, *D. simulans*, and *D. yakuba* within two linked regions encompassing partial sequences of *lbe* and *lbl*. The evolution of *lbe* and *lbl* is highly constrained due to their important regulatory functions. Despite this, a variety of forces have shaped the patterns of variation in *lb* genes: recombination, intragenic gene conversion and natural selection strongly influence background variation created by linkage disequilibrium and dimorphic haplotype structure. The two genes exhibited similar levels of nucleotide diversity and positive selection was detected in the noncoding regions of both genes. However, synonymous variability was significantly higher for *lbe*: no nonsynonymous changes were observed in this gene. We argue that balancing selection impacts some synonymous sites of the *lbe* gene. Stability of mRNA secondary structure was significantly different between the *lbe* (but not *lbl*) haplotype groups and may represent a driving force of balancing selection in epistatically interacting synonymous sites. Balancing selection on synonymous sites may be the first, or one of a few such observations, in *Drosophila*. In contrast, recurrent positive selection on *lbl* at the protein level influenced evolution at three codon sites. Transcription factor binding-site profiles were different for *lbe* and *lbl*, suggesting that their developmental functions are not redundant. Combined with our previous results on nucleotide variation in esterase and other homeobox genes, these results suggest that interplay of balancing and directional selection may be a general feature of molecular evolution in *Drosophila* and other eukaryote genomes.

## Introduction

Genetic changes in the genes that encode transcription factor (TF) proteins can produce fundamental phenotypic differences between species [for review, see 1–4]. Moreover, changes in TF coding sequences can result in profound modifications of the body plan [5], [6]. In order to understand how complex phenotypes evolve, we need to understand how genes involved in transcriptional regulation evolve. A global genomic approach has revealed general trends in gene evolution and showed that positive Darwinian selection is an important factor driving molecular evolution [e.g., 7–9). An important limitation of large-scale genomic studies is that they were unable to identify small-scale, within-gene variation that may directly influence protein function and corresponding phenotypic characteristics. Also, whole genome approaches are unable to reveal population level variation necessary for a better understanding of TF sequence evolution. Quantification of segregating variation within populations at TF loci is necessary to infer selective pressures and to ascertain the functional effects of naturally occurring allelic variation and sequence divergence among orthologs.

The available data on between-species TF variation indicate high rates of sequence evolution among regulatory genes [e.g., 10,11]. Studies of intra-specific nucleotide variation in *Drosophila* have revealed that regulatory genes tend to be less polymorphic than structural genes [12]. In contrast, homeobox genes from the 93DE cluster of *D. melanogaster* exhibit high sequence variation in *bagpipe* (but not in adjacent *tinman*) genes [13]. Also the TFs involved in olfactory pathways in *Caenorhabditis* exhibited more between- and within-species variation than structural chemosensory genes [14]. These data indicate that even adjacent regulatory genes can differ greatly in the level and pattern of sequence variation. This suggests that different members of a regulatory gene cluster may be subject to distinct evolutionary forces [15].

Here we focus on two homeobox genes, *ladybird early* (*lbe*) and *ladybird late* (*lbl*), tandemly-arranged paralogs in *Drosophila*. We analyze the level and pattern of the *lbe* and *lbl* segregating nucleotide variation in natural populations and divergence between close *Drosophila* species in attempt to reveal evolutionary forces governing the evolution of these genes. Both *lbe* and *lbl* genes are members of the NK homeobox gene family that consists of closely linked interacting regulatory genes (in 5′ to 3′ order: *tinman* (*tin*), *bagpipe* (*bap*), *lbe*, *lbl*, *C15*, and *slouch* (*S59*)), located on the right arm of *D. melanogaster* chromosome 3 at cytological map position 93DE [16], [17]. The coding region of *lbe* is 4,124 bp long and consists of two exons (1,008 and 432 bp) and one intron (2,684 bp). The *lbe* gene encodes a protein of 479 amino acids: the *lbe* homeodomain is located within exon II. The *lbl* coding region is 23,419 bp long and consists of three exons (702, 180, and 237 bp) and two introns (22,012 and 571 bp). The *lbl* gene is alternatively spliced, with three different LBL protein isoforms deduced from the sequence of cDNA clones [18], consisting of 342, 372, and 411 amino acids. The first part of the *lbl* homeodomain is in exon II and the rest in exon III, with a 571-bp intron that interrupts the *lbl* homeodomain. The transcription start site of *lbl* is 8.0 kb downstream of the *lbe* terminal stop codon. No ORF has been recorded in the region between the two genes and both genes are transcribed from the same DNA strand. The two genes show high similarity in the regions extending downstream and immediately upstream from the homeodomain [18], [19]. The deduced LBE and LBL amino acid sequences are 97% identical in the homeodomain, 61–81% identical in the upstream conserved region and 77% identical downstream of the homeodomain [17].

The *lbe* and *lbl* genes encode transcription regulators, which play an important role in neurogenesis, myogenesis, and cardiogenesis [17], [18], [20]. These genes have almost identical expression patterns, although *lbe*, located at the 5′ end, is activated slightly earlier than *lbl*; another difference concerns the trunk epidermis, where *lbe* transcripts are much more abundant [18]. Based upon their similar amino acid composition and expression patterns, both genes are often jointly referred to as “*ladybird* (*lb*)” [17], [20]. Analyses of *lb* gain-of-function phenotypes and rescue experiments have led to the conclusion that *lbe* and *lbl* are functionally redundant [17], [18]. In addition to *Drosophila*, *lb*-like genes have been detected in the sponge *Sycon raphanus* [21] and the mollusk *Loligo opalescens* [22], and orthologous genes have been found in mouse, chicken, and human genomes [23]–[25]. It is currently thought that the ladybird genes have an evolutionarily conserved role in development.

We previously investigated nucleotide variability in the *tin* and *bap* homeobox genes, located on the right arm of chromosome 3 of *D. melanogaster* within the 93DE cluster [13]. We now analyze nucleotide variation in *lbe* and *lbl* homeobox genes in 70 strains of *D. melanogaster* in four populations from East Africa (Zimbabwe), Europe (Spain), North (California) and South (Venezuela) America. We sequenced 4,482 bp covering the homologous coding (including the homeodomain) and noncoding (intron and 3′-flanking) regions for both genes (2,044 bp for *lbe* and 2,438 bp for *lbl*). The *lbe* and *lbl* genes display distinctive transcription factor binding-site profiles, suggesting that they are not redundant in developmental function. Negative selection and demography are major factors shaping the pattern of nucleotide polymorphism in the two genes. However there are clear indications of positive selection in the coding and noncoding regions of both genes, as well as balancing selection at synonymous sites in the *lbe* gene.

## Results

### Nucleotide Polymorphism

We detected similar total nucleotide diversity for *lbe* and *lbl* (Tables 1 and 2) close to the levels observed for *tin* and *bap* from the same 93DE gene cluster [13]. These estimates were within the range found in highly recombining gene regions of *D. melanogaster* [12] and in other regulatory genes [e.g., 15]. Figures S1 and S2 show the polymorphisms observed in 70 lines of *lbe* and *lbl*, including length polymorphisms. No nonsynonymous variability was detected in the *lbe* gene, but three nonsynonymous polymorphic sites were found in *lbl* (exon III, Fig. S2). While silent nucleotide diversity for *lbe* and *lbl* was similar (Tables 1 and 2), the level of synonymous polymorphism was 4.9 times higher in *lbe* than *lbl* (*P*<0.001). Synonymous variability of *lbe* was 4.4 times higher than noncoding variation (Table 1), a difference statistically significant in simulations even without recombination (*P* = 0.01), but the observed difference was not significant for *lbl*. There were five polymorphic sites within the *lbe* homeodomain (positions 1045, 1055, 1058, 1088, and 1121, Fig. S1), with π = 0.0086, slightly higher than for the whole *lbe* coding region (π = 0.0057). In contrast, there were two polymorphic sites in the *lbl* homeodomain (exon II, positions 837 and 903, Fig. S2), with π = 0.0013.

**Table 1:** Nucleotide diversity and divergence in the *lbe* gene region of *D. melanogaster*.

		*lbe* exon II					Full sequence	
	Intron I	Syn	Nsyn	Total	3′-fl. region	Ncod	Silent	All sites
N	948	100	329	429	568	1516	1616	1945
S	21 (7)	8 (0)	0 (0)	8 (0)	23 (5)	44 (12)	52 (12)	52 (12)
π	0.0035	0.0244	0	0.0057	0.0090	0.0055	0.0067	0.0056
θ	0.0046	0.0166	0	0.0039	0.0084	0.0060	0.0068	0.0056
*K_mel-sim_*	0.0257	0.1425	0	0.0309	0.0632	0.0393	0.0454	0.0374
*K_mel-sec_*	0.0330	0.1425	0	0.0309	0.0705	0.0469	0.0526	0.0433
*K_mel-yak_*	0.0792	0.1791	0	0.0382	0.1333	0.0986	0.1035	0.0845

Note. — Calculations based on 70 *D. melanogaster* lines from three populations: Barcelona, El Rio (California) and Venezuela, plus three lines from Zimbabwe and one *lbe* sequence from GenBank (accession number NT_033777.2). N, number of sites (indels excluded); S, polymorphic sites (number of singletons in parentheses); π, average number of nucleotide differences per site among all pairs of sequences [104, p. 256]; θ, average number of segregating nucleotide sites among all sequences, based on the expected distribution of neutral variants in a panmictic population at equilibrium [105]; *K_mel-sim_*, *K_mel-sec_*, and *K_mel-yak_* refer to nucleotide differences between *D. melanogaster* and *D. simulans*, *D. sechellia* or *D. yakuba*, respectively; Syn, synonymous sites; Nsyn, nonsynonymous sites; Ncod, noncoding (intronic and flanking regions); Silent, silent sites (synonymous and noncoding sites).

**Table 2:** Nucleotide diversity and divergence in the *lbl* gene region of *D. melanogaster.*

		*lbl* exon II + exon III						Full sequence	
	Intron I	Syn	Nsyn	Total	Intron II	3′-fl. region	Ncod	Silent	All sites
N	725	94	311	405	264	643	1632	1726	2037
S	16 (3)	3 (1)	3 (0)	6 (1)	24 (6)	25 (11)	65 (20)	68 (21)	71 (21)
π	0.0053	0.0049	0.0025	0.0031	0.0220	0.0069	0.0086	0.0084	0.0075
θ	0.0046	0.0066	0.0020	0.0031	0.0189	0.0081	0.0083	0.0082	0.0072
*K_mel-sim_*	0.0357	0.0585	0.0099	0.0209	0.1200	0.0674	0.0599	0.0598	0.0517
*K_mel-sec_*	0.0299	0.0760	0.0112	0.0257	0.1223	0.0781	0.0623	0.0631	0.0547
*K_mel-yak_*	0.0736	0.1496	0.0140	0.0452	0.2772	0.1885	0.1411	0.1416	0.1186

See Table 1, Note.

Synonymous variation of *lbe* was higher than in *Est-6* and ψ*Est-6* (for the same population samples), which were among the most polymorphic genes in *D. melanogaster* [26], [27]. High synonymous variability within *lbe* exon II was associated with the highest level of pair-wise divergence between *D. melanogaster* and three other *Drosophila* species (Table 1), which was several times higher than the divergence of the noncoding region (0.143–0.179 *vs*. 0.039–0.099). Functional significance could account for the fixation of favored codons, increasing the synonymous divergence in *lbe* exon II. The high variability of *lbe* exon II cannot be accounted for by relaxation of functional constraints, since it contains a functionally important homeodomain (180 bp at position 999–1178, our coordinates), which is conserved within a wide phylogenetic scale [17].

Variability of *lbl* exon III (π = 0.0045) was 3.5 times higher than exon II (π = 0.0013); a significant difference (*P*<0.01) in coalescent simulations. Increased total variability within *lbl* exon III was accompanied by decreased silent divergence between *D. melanogaster* and *D. simulans*, which was 4.3 times lower in exon III than in exon II (0.0237 *vs.* 0.1020). Relaxation of functional constraints is one possible explanation for the prevalence of replacement substitutions in *lbl* exon III. However, these patterns indicated that the *lbl* coding region was under strong negative selection (see below), possibly imposed by alternative splicing [28] described for this gene [17]. Elevated replacement substitutions may indicate a functional shift of the *lbl* coding region evolving under positive selection. Below, we used neutrality tests and codon models to test this hypothesis. There was also a significant difference in population variability between *lbl* introns, 4.2 times higher in intron II than in intron I (π = 0.0220 *vs*. 0.0053, Table 2). A parallel difference in species divergence was observed between these two introns (see *K_mel-sim_* and *K_mel-sec_* in Table 2). The elevated divergence in the *lbl* intron II is puzzling. The region is rich with transcription factor binding sites (see below the section “Binding Site Profile”) and its complex architecture might be related to the specific evolutionary dynamic of intron enhancers that can evolve beyond recognizable sequence similarity while retaining function [e.g., 29]. For more details concerning nucleotide polymorphism, see Text S1 and Tables S1, S2, S3.

### Recombination and Gene Conversion

The method of Hudson and Kaplan [30] revealed a minimum of 10 recombination events for *lbe*, 14 for *lbl*, and one between them. Estimates of the recombination rate *ρ* and the *ρ*/θ ratio were higher for *lbl* than for *lbe* (*ρ* = 0.016 and 0.006, respectively; Table S4). Previously we found a large difference (∼33 times) in recombination rate between *tin* (*ρ* = 0.001) and *bap* (*ρ* = 0.026), within the 93DE cluster [13]. For *esterase* genes on the left arm of *D. melanogaster* chromosome 3 at cytological map position 68F7-F8, the rate was nearly three times higher for *Est-6* than for ψ*Est-6* (0.021 *vs.* 0.008) [26], [27]. This suggests that noticeably different recombination rates are common in tandemly associated paralogs. The recombination rate was similar for European and North American samples, but much lower for South America (Table S4). A similar trend was observed for *Est-6* and ψ*Est-6* [26], [27].

Sawyer's method [31] detected gene conversion events within *lbe* (except Venezuela) and *lbl,* but the number of significant events was considerably higher for *lbl* (Table S5). The average fragment length was 1,202 bp for *lbe* but 703 bp for *lbl*. Previously, we observed similar differences in average fragment length for *tin* (1,396 bp) and *bap* (665 bp) [13]. There was no evidence of intergenic gene conversion, likely due to low nucleotide similarity between *lbe* and *lbl* (53.5%), insufficient for efficient intergenic conversion.

### Haplotype Structure

**Figure 1:**
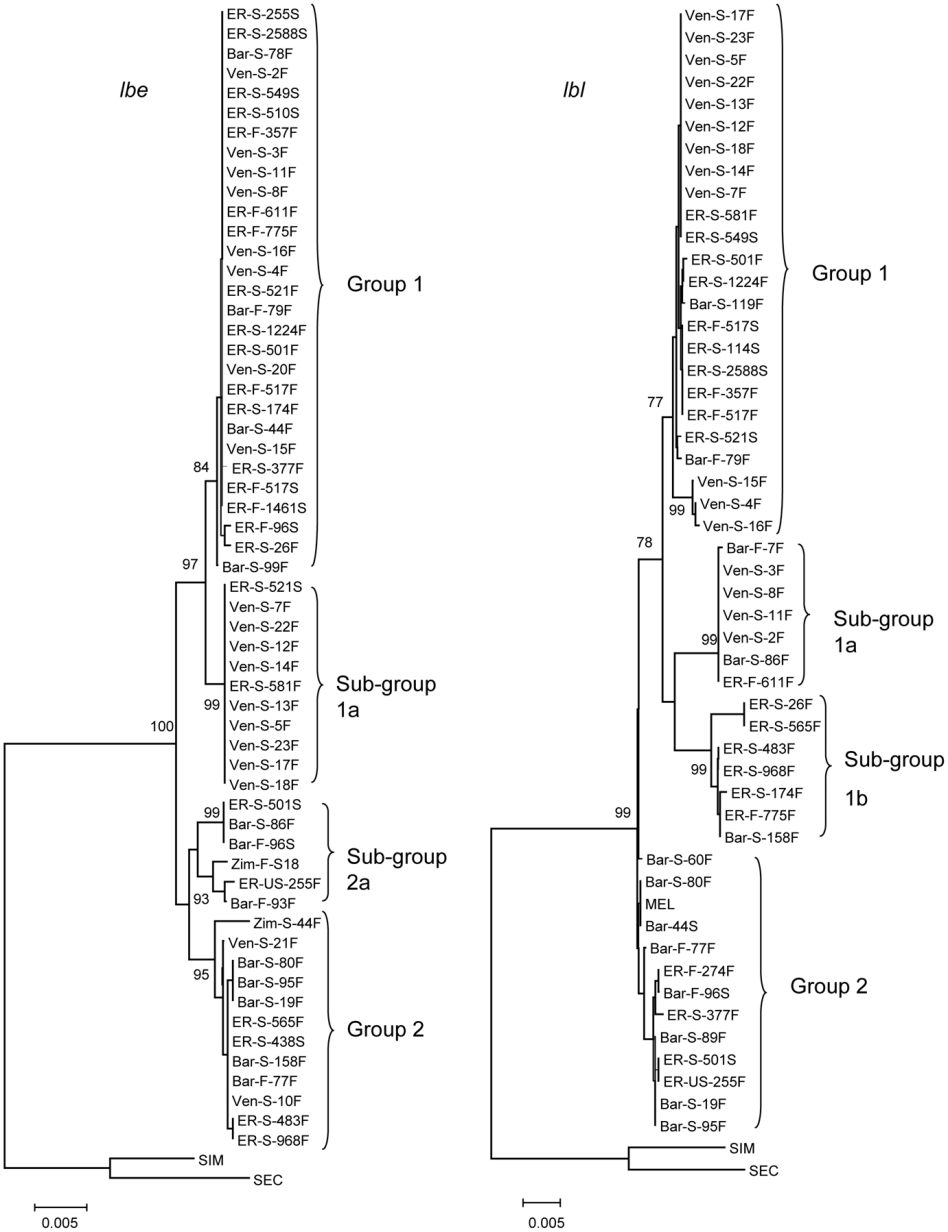
Neighbor-joining tree of *lbe* (left) and *lbl* (right) haplotypes of *D. melanogaster,* excluding recombinant sequences. The tree is based on Kimura 2-parameter distance. Numbers at the nodes are bootstrap percent support values based on 10,000 replications. Strains as in Fig. S1. Haplotype groups are encompassed in brackets. SEC, *D. sechellia*; SIM, *D. simulans.*

The *lbe* haplotype structure (excluding recombinants, see Fig. S1) is shown in Fig. 1, left. Due to recombination and gene conversion, this tree is not good reflection of the genealogical process, but serves to show the genetic structure of the data. There were two main *lbe* haplotype groups (1 and 2 in Fig. 1) and two sub-groups (1a and 2a). The main haplotype groups exhibited 19 nucleotide differences: 14 fixed within noncoding regions and five almost fixed within coding regions (excepting two recombinant variants detected for Ven-S-21F and Zim-S-44F; Fig. S1). The groups were differentially associated with indels. Group 2 was completely associated with three deletions (1-, 4-, and 30-bp, within intron I and the 3′-flanking region; ▴1, ▴4, and ▴6, Fig. S1). Group 1 was associated with two polymorphic indels (22-bp insertion and 8-bp deletion within the 3′-flanking region; ▾1 and ▴7; Fig. S1). These five indels were not found in the *lbe* sequences of *D. simulans* and *D. sechellia*, suggesting that they may represent a derived condition after the split of *D. melanogaster* from the other two species. The difference between the two *lbe* haplotype groups was highly significant (F_st_ = 0.84; *K*_st_ = 0.75; *P*<0.001).

There were two *lbe* haplotype sub-groups (1a and 2a); related respectively to group 1 and to group 2 (Fig. 1, left). Sub-group 1a had seven nearly fixed differences from group 1 (excepting one recombinant variant, ER-S-26F), all within noncoding regions (Fig. S1). The coding regions were identical for all *lbe* group 1. Sub-group 2a differed from group 2 by nine fixed nucleotide differences (excepting one recombinant variant, Zim-S-44F), all within the 3′-flanking region. There were six polymorphic sites within the coding region with different frequencies for group 2 and sub-group 2a; however, none of these differences were fixed. Sub-group 2a was associated with two indels (2-bp deletion and 10-bp insertion within the 3′-flanking region; ▴8 and ▾2; Fig. S1).

Strong haplotype structure was also observed for *lbl*, with two main haplotype groups (1 and 2), and two sub-groups, 1a and 1b (Fig. 1, right). The *lbl* haplotype groups were differentially associated with indels: sub-group 1a was fully associated with a 12-bp insertion and a 26-bp deletion within intron II (▾2 and ▴2, Fig. S2); sub-group 1b was associated with a 9-bp deletion within exon III (▴6); group 2 was associated with two deletions (78- and 237-bp long) and a 24-bp insertion (▴1, ▾3, and ▴3); there were no indels within group 1. The difference between the *lbl* haplotype groups was highly significant (F_st_ = 0.86; *K*_st_ = 0.54; *P*<0.001). Total sequence divergence (*D*_xy_) among the *lbl* haplotype lineages was 0.0106 (ignoring indels), similar to *lbe* (0.0081).

Group 1 *lbe* haplotypes were most frequent in our data set, but variability was low (π, total  = 0.0002), 5.5 times lower than in group 2. Group 2 haplotypes were less frequent and more variable (π, total  = 0.0011). Group 2 is likely the ancestral state, consistent with the higher polymorphism of group 2 haplotypes, and supported by the comparison with *D. simulans* and *D. sechellia*. Group 1 may have evolved under directional selection (high frequency and low variability haplotype profile). For the North American sample (excluding recombinants), there were 37 polymorphic sites, and there was a subset of 17 sequences (haplotype group 1) with only four polymorphic sites (Fig. S1). The haplotype test [32] was significant (*P* = 0.02) with recombination rate *ρ* = 0.015. For the South American sample, the test was also significant (*P*<0.01), as it was significant for the total dataset, contrasting group 1 with all available sequences (*P* = 0.02). The haplotype test was not significant for the full length of the *lbl* gene region, which could be explained by elevated number of polymorphic sites within intron I and 3′-flanking region (Fig. S2). However the test was significant (*P* = 0.025) for the *lbl* region including exon II, intron II, and exon III for the total dataset. Thus, the main haplotype group 1 might evolve under the influence of directional selection in both the *lbe* and *lbl* genes. For more details concerning haplotype structure, see Text S2.

### Sliding Window Analysis

**Figure 2:**
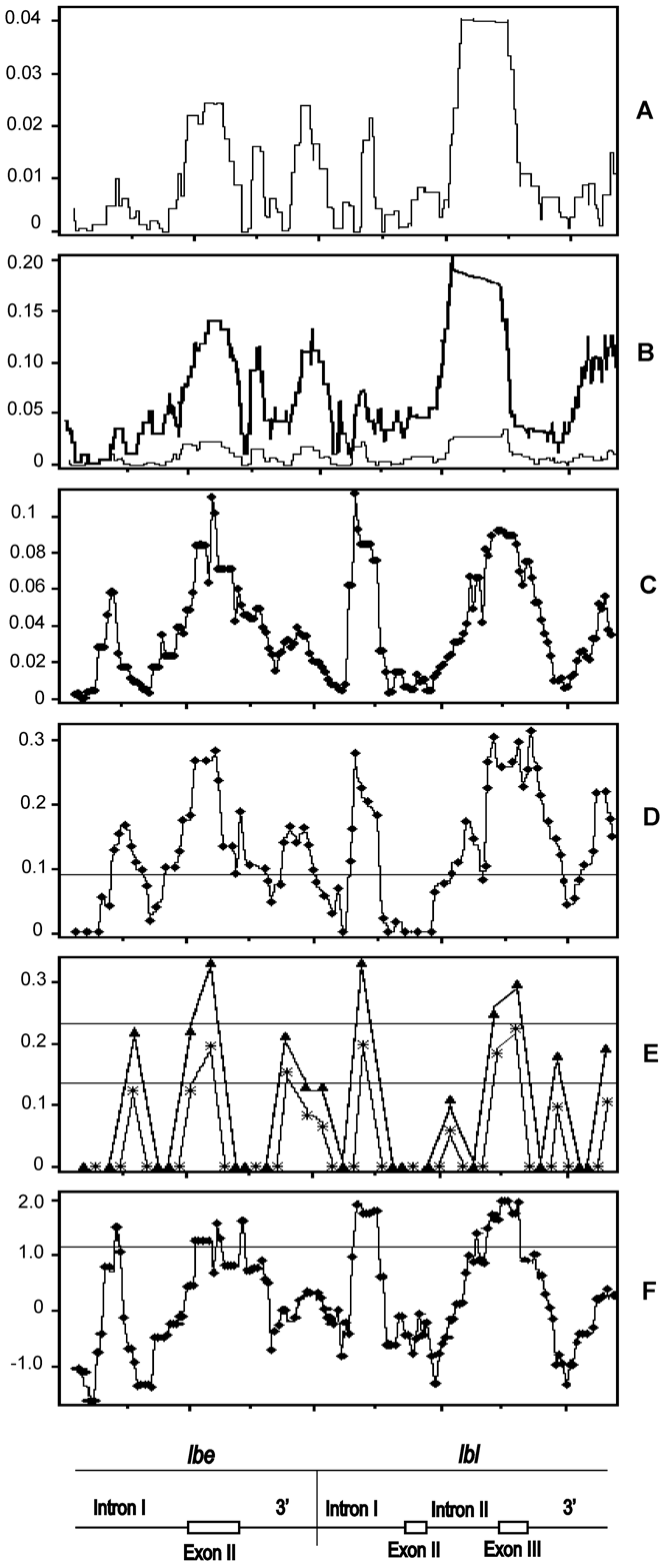
Sliding window plots along the *lbe* and *lbl* genes of *D. melanogaster*. A, silent nucleotide diversity; B, silent nucleotide diversity (thin line) and divergence (thick line); C, linkage disequilibrium measured by *D*; D, E, and F, neutrality test statistics of Kelly's *Z_nS_* [33], Wall's *B* and *Q* [34], and Tajima's *D* [35], respectively. Window sizes are 100 nucleotides with one-nucleotide increments for A and B; 250 nucleotides with 25-nucleotide increments for C; 250 nucleotides with 30-nucleotide increments for D and F; and 250 nucleotides with 150-nucleotide increments for E. A schematic representation of the region investigated is displayed at bottom. 99% confident intervals for neutrality test statistics (D, E, and F plots) are marked by thin horizontal lines (there are two horizontal lines in E: Top is for the *B* statistic and bottom is for the *Q* statistic of Wall's [34] test) obtained by coalescent simulations conditioned on the number of polymorphic sites with the recombination rate equal to 0.015.

There were noticeable peaks of nucleotide variability along the *lbe* and *lbl* genes (Fig. 2). For *lbe*, bursts of variability were observed for intron I, exon II, and the 3′-flanking region with the most pronounced peak of silent polymorphism in exon II (midpoint coordinates 1073–1254; Fig. 2A). For *lbl*, there were three significant peaks within noncoding regions with the most pronounced peak observed for intron II, 3 – 4 times more polymorphic than other noncoding regions of *lbl*. The coding region of *lbl* also showed a significant peak of nucleotide variability in exon III accompanied by decrease of divergence that strongly contrasted with the high divergence in the adjacent regions (Fig. 2B). Peaks of nucleotide diversity were accompanied by increased levels of linkage disequilibrium (Fig. 2C) and significant values of Kelly's [33], Wall's [34], and Tajima's [35] neutrality test statistics (Fig. 2D, E, and F), suggesting that positive (balancing or diversifying) selection may overcome the dominant effect of negative selection in this highly constrained functional region.

Patterns of polymorphism and divergence were different in *lbe* and *lbl*. For *lbe* the highest silent polymorphism and divergence were observed in exon II, but for *lbl* in a noncoding region, intron II. We suggest that there may be multiple targets of positive selection within the noncoding and coding regions of both genes. Despite the absence of replacement substitutions in *lbe*, the most pronounced *lbe* peak of variability was located within exon II, seven and three times more polymorphic than intron I and the 3′-flanking region, respectively. One explanation is that balancing selection acts on synonymous sites of *lbe* (additional support for this premise below).

Two types of selection might be involved in the evolution of the *lb* genes: balancing selection that creates elevated nucleotide variation around target polymorphic sites, and directional selection that creates significant excesses of very similar sequences exhibiting very low levels of variation. A similar scenario was inferred to explain patterns of nucleotide polymorphism for *Est-6* and *bagpipe* in *D. melanogaster* [13], [26], [36].

### Tests of Neutrality and Maximum Likelihood Analysis of Selective Pressures

Neutrality tests detected significant deviations from neutrality in the *lb* region (Table S7), based on linkage disequilibrium (LD) between segregating sites with recombination (*ρ* = 0.015, see above). When applied to the full *ladybird* gene region, Kelly's [33] *Z_nS_* and Wall's [34] *B* and *Q* tests were highly significant for the whole dataset, and for each population separately (Table S7). The tests were also significant separately for the *lbe* and *lbl* genes (except the European sample) as well as for the coding and flanking regions of both genes (Table S7). Significant values of Kelly's and Wall's statistics were grouped around the peaks of linkage disequilibrium (Fig. 2), and centered around the coding and noncoding regions of *lbe* and *lbl*, which supporting the hypothesis that these sites were targets of balancing selection. For more detailed information concerning LD, see Text S3, Fig. S3, and Table S6.

However, neutrality tests are typically affected by demography and so may be difficult to interpret [37], [38]. We applied model-based maximum likelihood (ML) methods to confirm the observations made above (results summarized in Table 3). All results from the ML analyses revealed below held for the full sample and for the *D. melanogaster* strains separately, whether or not recombinant sequences were removed.

**Table 3:** Likelihood ratio tests LRTs based on codon models of evolution.

LRTs null *vs.* alternative	Genes^a^	LRT statistic^b^	*P*-value^b^	Estimates of interest^c^
M0 *vs.* M3	*lbe*	0	1	ω = 0, *κ* = 2.09, *t* = 0.37, *d*_S_ = 0.80, *d*_N_ = 0
	*lbl*	45.37	**<0.001**	ω = 0.22, *κ* = 3.72, *t* = 0.27, *d*_S_ = 0.22, *d*_N_ = 0.05
	*Est-6*	164.50	**<0.001**	ω = 0.23, *κ* = 3.14, *t* = 0.44, *d*_S_ = 0.33, *d*_N_ = 0.08
	ψ*Est-6*	184.62	**<0.001**	ω = 0.29, *κ* = 1.64, *t* = 0.61, *d*_S_ = 0.44, *d*_N_ = 0.13
	*bap*	50.43	**<0.001**	ω = 0.10, *κ* = 4.77, *t* = 0.22, *d*_S_ = 0.24, *d*_N_ = 0.02
	*tin*	0	1	ω = 0.13, *κ* = 3.23, *t* = 0.12, *d*_S_ = 0.13, *d*_N_ = 0.02
M3* *vs.* Dual	*lbe*	54.64	**<0.001**	CV_S_ = 3.59, CV_N_ = 0
	*lbl*	19.47	**<0.001**	CV_S_ = 2.27, CV_N_ = 6.00
	*Est-6*	130.38	**<0.001**	CV_S_ = 2.29, CV_N_ = 4.42
	ψ*Est-6*	132.06	**<0.001**	CV_S_ = 2.05, CV_N_ = 2.13
	*bap*	28.08	**<0.001**	CV_S_ = 2.33, CV_N_ = 5.77
	*tin*	1.08	0.90	CV_S_ = 0, CV_N_ = 0
FMutSel0 *vs.* FMutSel	*lbe*	101.37	**<0.001**	P_+_ = 0.22, S_+_ = 0.73, S_–_ = – 3.53
	*lbl*	75.21	**<0.001**	P_+_ = 0.04, S_+_ = 2.27, S_–_ = – 4.60
	*Est-6*	128.93	**<0.001**	P_+_ = 0.25, S_+_ = 0.83, S_–_ = –1.47
	ψ*Est-6*	109.21	**<0.001**	P_+_ = 0.31, S_+_ = 0.64, S_–_ = – 0.96
M7 *vs.* M8	*lbl*	21.14	**<0.001**	*p* = 8.84, *q* = 99, *p*_0_ = 0.978, *ω* = 24.52, *p*_1_ = 0.022
	*Est-6*	63.15	**<0.001**	*p* = 0.33, *q* = 2.24, *p*_0_ = 0.986, *ω* = 9.20, *p*_1_ = 0.014
	ψ*Est-6*	27.21	**<0.001**	*p* = 0.01, *q* = 0.03, *p*_0_ = 0.994, *ω* = 9.05, *p*_1_ = 0.006
	*bap*	11.47	**0.003**	*p* = 0.01, *q* = 0.13, *p*_0_ = 0.992, *ω* = 9.90, *p*_1_ = 0.008
Selection on noncoding regions ζ = 1 *vs.* ζ≥1	*lbe*	178.86	**<0.001**	*ζ*_0_ = 0.20, *p*_0_ = 0.95, *ζ*_1_ = 6.55, *p*_1_ = 0.05
	*lbl*	25.11	**<0.001**	*ζ*_0_ = 0.08, *p*_0_ = 0.86, *ζ*_1_ = 2.81, *p*_1_ = 0.14

M3* is the HYPHY implementation of model M3 [98]. Tests comparing M3* *vs.* Dual and M7 *vs.* M8 were performed only for genes where M0 *vs.* M3 was significant. The test FMutSel0 *vs*. FMutSel was performed only for genes with large samples.

a Results for *lb* genes are shown for full samples. However, all LRT results remain the same when only *D. melanogaster* was analyzed (with or without the recombinants).

b LRT statistic is double the difference between the likelihood values optimized under the alternative and null models: 2(???_alt_ – ???_null_). *P*-value is computed using the χ^2^-distribution with d.f. = 4 for LRTs of M0 *vs.* M3, and M3* *vs.* Dual. For LRT of M7 *vs.* M8 d.f. = 2, and for FMutSel0 *vs.* FMutSel d.f. = 41. For the LRT of positive selection on noncoding regions d.f. = 1. Significant *P*-values in bold.

c Estimates under M0: ω = *d*_N_/*d*_S_; tree length *t* is measured by number of expected nucleotide substitutions per codon over the tree; transition/transversion ratio κ (estimated under M3, or under M0 if the test M0 *vs.* M3 is not significant); *d*_S_, tree length for synonymous substitutions , *d*_N_, tree length for nonsynonymous sites. Estimates under Dual model: coefficients of variation ( = standard deviation/mean) for distributions of synonymous and nonsynonymous rates, CV_S_ and CV_N_ respectively. Estimates under mutation-selection model FMutSel: P_+_ is the proportion of mutations with advantageous effect (*S* = 2*Ns*>0); S_+_ is mean selection coefficient of all advantageous mutations; S_–_ is mean selection coefficient of all deleterious mutations. Estimates under M8: *p*, *q* are parameters controlling the shape of the Beta-distribution; *p*_0_ is proportion of sites in the sequence with *ω*-values from beta-distribution (between 0 and 1); *ω* = *d*_N_/*d*_S_, with discrete class allowed to be >1 (under positive selection); *p*_1_ is proportion of sites in the discrete class. In the combined coding and noncoding model [102], sites in the noncoding region come from two categories: proportion *p*_0_ are under negative selection with *ζ*_0_<1, and *p*_1_ are under positive selection or evolving neutrally with *ζ*_1_≥1; *ζ*_0_ and *ζ*_1_ are estimates of the ratio of substitution rate in the noncoding region to average synonymous rate in the coding region for the two categories.

The test for variability of diversifying selection on the protein was not significant for *lbe* (M0 *vs.* M3) [39] as stringent purifying selection prohibited nonsynonymous changes. In contrast, this test was highly significant for *lbl* (Table 3). The test for positive selection (M7 *vs.* M8, Table 3) was significant for *lbl*, suggesting that 2% of sites evolved under strong diversifying selection (ω≈24.5), while the remaining sites were very conserved (ω≈0.1). Sites 118Q, 122S and 131L (in bold in Fig. S2) in *lbl* were subject to positive selection at the protein level (posterior probability >0.99). Lower, but significant, levels of positive selection were detected in other genes (Table 3), with 7, 3, and 1 sites under positive selection, respectively for *Est-6*, ψ*Est-6*, and *bap* (posterior probability >0.99), consistent with previous results [13], [40].

The synonymous rate *d*_S_ in the coding sequence varied significantly for both *lbe* and *lbl* (M3 *vs.* M3-Dual in Table 3). Coefficient of variation (CV) was used to compare the variability of nonsynonymous and synonymous rates estimated under the M3-Dual model. Synonymous variation was higher in *lbe* with CV_S_ = 3.59, compared to 2.27 in *lbl*. Interestingly, the synonymous variation in *lbl* was similar to that in other genes, such as *Est-6*, ψ*Est-6* and *bap* with CV_S_ = 2.29, 2.05, and 2.33, respectively. In these genes, the nonsynonymous rate variation (CV_N_) was much higher than for the synonymous rate, with CV_N_ 4.4 – 6 and CV_S_ 2 – 2.3. For ψ*Est-6* and *tin* the variation in *d*_N_ and *d*_S_ was similar. In contrast, synonymous variation was not only higher in *lbe* comparatively to other genes, but was accompanied by a total absence of nonsynonymous substitutions (CV_N_ = 0). Seven percent of sites in *lbe* evolved with unusually high *d*_S_ = 14.3, whereas at the remaining sites the rate was constrained to *d*_S_ 0.01 – 0.08.

Widespread positive selection in synonymous sites was previously reported for mammalian genes [41] where selection may act through mRNA destabilization affecting mRNA levels and translation [42], [43]. Since protein folding is thought to occur simultaneously with protein translation from mRNA, the use of preferred and unpreferred codons may affect protein translation rates [44]. All polymorphic sites in the *lbe* coding region (Fig. S1) showed anomalously high rates of synonymous substitutions (posterior probabilities >0.99). These sites may be responding to diversifying selection on synonymous codons, perhaps affecting the speed of translation, with possible implications for protein folding (see below “mRNA Secondary Structure Stability” for details).

Synonymous codon usage is typically high in *Drosophila* [e.g., 45,46]. In our data, the test comparing selection-mutation models FMutSel0 *vs*. FMutSel was highly significant for both *lb* genes and all other members of the 93DE gene cluster (Table 3), suggesting that natural selection was a driving force in the evolution of synonymous codons.

Finally, the LRT for positive selection in noncoding regions (see "Materials and Methods") was significant for both *lb* genes (Table 3). For *lbe*, 5% sites in the noncoding region were estimated to evolve by positive selection, with a substitution rate more than six times higher than the average *d*_S_ in the coding region of *lbe* (ζ_1_ = 6.55). For *lbl*, a larger proportion of sites (14%) was estimated to be under positive selection, although the estimated selection pressure was lower, with ζ_1_ = 2.81. Such differences in estimated positive selection pressure on noncoding regions are especially striking considering that the average *d*_S_ is much higher for *lbe* than for *lbl* (estimates from M0, Table 3). For more detailed information concerning ML analyses, see Text S4.

### mRNA Secondary Structure Stability

We calculated RNA secondary structure free energy for the representative sequences of the main *lbe* and *lbl* haplotype groups (Table 4) using the program RNAstructure [47]. The major *lbe* haplotype groups (I and II, Fig. 1) significantly differed (*P* = 0.0001) with respect to mRNA secondary structure while the *lbe* group I haplotypes were less stable than group II. In contrast to *lbe*, the difference in mRNA stability between *lbl* haplotypes was small and not statistically significant (*P*>0.5). Predicted mRNA stability was much higher for *lbe* than for *lbl* (Mann-Whitney test *P*<0.0001) for both haplotype groups (Table 4).

**Table 4:** Free energy (ΔG, kcal/mol) of *lbe* and *lbl* mRNA base pairing.

	Group I (ER-S-2588S)		Group II (ER-S-565F)		
	Mean	St. dev	Mean	St. dev	*P*
*lbe*	−144.639	1.6323	−146.734	2.0640	0.0001
*lbl*	−90.061	2.4110	−89.676	2.3594	N.S.

St. dev.: standard deviation.

Thus, *lbe* haplotypes divergent in only synonymous changes exhibited significant differences in mRNA stability. This observation highlights the potential significance of *lbe* synonymous variation, providing indirect evidence for the functional basis of balancing selection maintaining synonymous variation in this gene. Given the evidence from other studies that differences in mRNA secondary structure stability can affect mRNA decay [48], gene expression [49], [50], and level of protein translation [51], [52], we propose that *lbe* synonymous polymorphisms may be important contributors to adaptive variation in *D. melanogaster*. Because there was no difference in secondary structure stability between *lbl* mRNA transcripts representing two main haplotype groups, mRNA secondary structure for this gene may not be a target for positive selection. We showed above (section “Tests of Neutrality and Maximum Likelihood Analysis of Selective Pressures”) that for the *lbl* gene, positive selection operated on the protein level.

Predicted mRNA stability was much higher for *lbe* than for *lbl* (Table 4; Mann-Whitney test *P*<0.0001), consistent with the GC difference between the genes. Total GC content was significantly higher in *lbe* than *lbl* (59.5% *vs.* 51.3%; Wilcoxon test *P* = 0.0001; Mann-Whitney test *P* = 0.0001). It was shown previously that increased levels of GC in coding sequences have a stabilizing effect on mRNA secondary structure [e.g., 53]. Accordingly, *lbe* evolution was associated with increased stability and balancing selection on mRNA secondary structure whereas *lbl* evolution was accompanied by lower mRNA stability and positive selection at the protein level.

### Binding Site Profile

*Drosophila* transcription factor motifs were obtained from FlyReg database curated motifs [54], and we used ClusterDraw2 [55] to scan for binding site matches and binding site clusters. The program uses a polynomial model to determine statistical significance of binding site clusters. The cluster significance cutoff was set to 3, corresponding to a significance level of *P* = 0.001. We analyzed 44 transcription factor motifs and detected significant clusters for 30 motifs (Table S8). The spatial distribution of clusters was not uniform along *lb* genes because the vast majority of significant clusters were located within non-coding sequences. The deviation from equal proportion of significant clusters was highly significant for both *lbe* (χ^2^ = 29.13, d.f. = 1, *P*<0.001) and *lbl* (χ^2^ = 32.30, d.f. = 1, *P*<0.001). Twelve of 30 motifs were significant for both genes. Ten clusters were significant only for *lbe* (part of a specific component of the *lbe* regulatory profile). Eight clusters were significant only for *lbl* (specific component of the *lbl* regulatory profile). In *lbe* and *lbl*, these clusters coincided for 40% of the binding sites, whereas the remaining clusters were gene-specific. The distribution of significant binding-site clusters for *lbe* and *lbl* was highly asymmetric, with a proportion of specific clusters >50%. The difference in binding-site profiles suggests that the genes are not redundant in developmental function.

## Discussion

Our analyses revealed a dimorphic haplotype structure for both *lb* genes. Despite similar levels of total nucleotide diversity in *lb* genes, synonymous nucleotide variability and the variation in the synonymous rate of change were much higher in *lbe* than in *lbl* and other genes from the 93DE cluster (*tin* and *bap*) as well as *Est-6* and ψ*Est-6* that are among the most polymorphic genes of *D. melanogaster* [13], [26], [27]. We attribute this high synonymous variation to balancing selection on *lbe* synonymous sites. Resch et al. [41] showed for mammalian genes that positive selection at synonymous sites may act through mRNA destabilization affecting mRNA levels and translation. This observation is in accordance with widespread compensatory evolution at the molecular level, caused by epistatic selection maintaining mRNA secondary structures [56]. The mechanism underlying epistatic selection is based on a model of compensatory fitness interactions [57], which suggests that mutations in RNA helices are individually deleterious but become neutral in appropriate combinations. The presence of significant excess of synonymous variation and clear influence of this variation on mRNA secondary structure stability suggests adaptive compensatory evolution in the *lbe* gene.

There are numerous studies devoted to synonymous site evolution in *Drosophila* [recent reviews in 43,58-60]. The main focus of these investigations is codon usage bias where different synonymous codons are used with different frequencies. It was shown that codon usage is tuned to optimize for expression and is adapted to tRNA pools of the organism. This is a type of purifying selection on synonymous sites preserving the usage of optimal codons [e.g., 42,45,61].

Analysis of the *Notch* locus in *D. melanogaster* has identified a region with accelerated synonymous site divergence [62]–[64]. The authors found an excess of fixed unpreferred codons and concluded that directional selection on synonymous sites had driven the fixation of these unpreferred codons. Later Holloway et al. [65] detected similar patterns for 64 genomic elements, a majority of which reside in protein-coding regions in the *D. melanogaster* genome. A genome-wide computational analysis showed that some unpreferred codons were fixed by directional selection in both bacteria and flies [66], [67]. Thus selection on synonymous sites is not limited to the preferential fixation of mutations that enhance the speed or accuracy of translation because in some situations selection for unpreferred codons can impede translation efficiency. Neafsey and Galagan [66] found that regulatory genes are particularly likely to be subject to selection for unpreferred codon usage. They suggested that low translational efficiency can be favored by reducing expressional noise through regulatory cascades [66]. Holloway et al. [65] hypothesized that ribosomal pausing for proper protein folding is a more tenable mechanism for explaining the preferable fixation of unpreferred codons than the alternative of reducing translation efficiency. However it was demonstrated that translational initiation of the ribosome can locally destabilize secondary structures and move along the mRNA without any significant delays [68] suggesting that the protein conformation alone cannot explain non-uniformity in translation elongation.

On the inter-specific level, we also found prevalent fixation of unpreferred codons in the *lbe* exon II (in *D. melanogaster* – *D. simulans* or *D. sechellia* comparisons, six out of eight synonymous changes lead to unpreferred codons). This pattern could not be explained by changes in mutation rates and/or low levels of recombination (see the section "Tests of Neutrality and Maximum Likelihood Analysis of Selective Pressures"). It is reasonable to suggest that directional selection on synonymous sites has driven the fixation of these unpreferred codons as in case with *Notch* and some other loci in *Drosophila* [62]–[67]. However intra-specific patterns of synonymous site variability in the *lbe* exon II suggest the involvement of balancing selection maintaining two different forms of mRNA molecules. Using site-specific codon models [69], [70], specifically developed to analyze site-specific variation, we detected a few sites in the *lbe* exon II where, despite strong codon bias due to negative selection on the whole gene, we observed very high rates of synonymous change consistent with balancing selection on those precise sites. Thus we found a site-specific phenomenon that cannot be explained by the influence of codon usage bias alone because divergent *lbe* coding haplotypes could not be a by-product of the selection on codon bias. Our data indicate that different mechanisms are involved in evolution of synonymous sites in the *lbe* gene compared to loci that have recent acceleration synonymous site divergence like *Notch* and some others (see references above). We argue that *lbe* intra-specific synonymous polymorphism is due to balancing selection maintaining two mRNA forms that can provide necessary functional flexibility [51], [58].

We detected contrasting patterns in nonsynonymous variation and rates in the *lbe* and *lbl* genes. While nonsynonymous mutations in *lbe* are prohibited, supposedly due to their strong detrimental effects on the LBE protein, three nonsynonymous mutations observed in *lbl* are predicted to have occurred under the influence of recurrent diversifying selection on the LBL protein. The level of the *lbl* nonsynonymous variability and *d*_N_ rate variation was much higher than its level of synonymous variability. The excess of replacement substitutions cannot be due to relaxation of functional constraints, because this region contains the homeobox region, which is highly conserved on a wide phylogenetic scale [17]. Moreover, strong selective constraints on the *lbl* coding region may be imposed by the alternative splicing described for this gene [17]. We suggest that the *lbl* gene may pass through evolutionary periods of functional transformation marked by the prevalence of replacement substitutions within *lbl* exon III, against a background of intensive negative selection.

Thus the two *lb* homeobox genes show contrasting patterns of nucleotide and codon evolution. Moreover, we have found a highly asymmetric distribution of significant binding-site clusters, with >50 % of binding-site clusters specific for either *lbe* and *lbl*. The distinct binding-site profiles of *lbe* and *lbl* suggest that the genes are not redundant in developmental function. On the contrary, Jagla et al. [17], [18], [20], assert that *lbe* and *lbl* are functionally redundant.

We have previously suggested that the pattern of nucleotide variability of the *Est-6* and *bap* coding regions in *D. melanogaster* are shaped by the influence of both directional and balancing selection [13], [26], [36]. Here, directional selection accounts for the excess of nearly identical sequences, and balancing selection prevents the complete fixation of haplotypes and increases the level of nucleotide variation. The present data show that both type of selection are involved in *lbe* and *lbl* evolution of *D. melanogaster*. A similar account has been proposed for the *Adh* and *TFL1* loci of *Arabidopsis thaliana* [71], [72], the *Acp29AB* locus of *D. melanogaster* [73], the *Pan I* locus of Atlantic cod *Gadus morhua* [74], the MHC *DQB1* locus of marine mammals *Orcinus orca*, *Tursiops truncates*, and *T. aduncus* [75], and the human *AVPRIB* gene [76]. Therefore, the operation and interaction of balancing and directional selection appears to be a general feature of molecular evolution in *Drosophila* and other eukaryote genomes.

The interaction between selective and neutral processes, nevertheless, should be cautiously interpreted given the modest sample size of sequences and the relatively short sequence lengths [e.g., 77]. Moreover, non-selective factors such as demography could partly account for the patterns of the polymorphisms. Demographic and selective forces shaping nucleotide polymorphism patterns in a species like *D. melanogaster* are difficult to disentangle because of its complicated history, including both recent worldwide migration and adaptation to drastically new environments [see, e.g., 78–80]. Patterns of polymorphism should be influenced by both of these evolutionary forces and is apparent in our data obtained for *Sod*, *Est-6*, ψ*Est-6*, *tin*, *bap*, *lbe*, and *lbl* located on the third chromosome from four natural *D. melanogaster* populations (Africa, Europe, North and South America). Comparative analysis showed significant peaks of variability in the *Est-6* region observed both in African and non-African samples, but dimorphic structure was detected only in non-African samples [26]. This observation supports the hypothesis that dimorphic haplotype structure is generated by demographic process during the recent history of *D. melanogaster* caused by admixture of differentiated populations\. Significant peaks of increased nucleotide variability accompanied by peaks of LD and centered on the functionally important sites may reflect the effects of balancing selection [13], [26], [36], [40], [81] – this hypothesis was predicted by theoretical analysis [82]–[86].

Each gene family has its own evolutionary history. Consequently, a full understanding of their evolution may require comprehensive data be obtained for all multigene families in the genome. Distinguishing between demography and selection, or establishing the relative importance of these evolutionary factors in the patterning of molecular variation may not be sufficient to achieve a deeper understanding of the whole nature of molecular variation evolving under multidirectional evolutionary forces. Consequently, future investigations are needed in other species and genes of *Drosophila* in order to resolve these problems.

## Materials and Methods

The *D. melanogaster* strains derive from wild flies collected in Europe (Barcelona, Spain; 19 strains), North America (California; 28 strains), and South America (Caracas, Venezuela; 19 strains). The strains were made fully homozygous for the third chromosome by crosses with balancer stocks, as described by Seager and Ayala [87]. Chung-I Wu kindly provided the isofemale *D. melanogaster* strains from East Africa (Sengwa, Zimbabwe). The three African strains included in our analysis were homozygous for the *lbe* and *lbl* gene regions. The strains used in the present study were previously investigated for the *β-esterase* gene cluster [26], [27], [36] and the homeobox genes *tin* and *bap* [13]. The *lb* sequences of *D. melanogaster*, as well as those of *D. sechellia*, *D. simulans*, and *D. yakuba* were obtained from GenBank (accession numbers: NT_033777.2, AAKO01001614, AAEU02001386, AAEU02001382, AAGH01024581, and AAKO01001614).

### DNA Extraction, Amplification, and Sequencing

Total genomic DNA was extracted using the tissue protocol of the QIAamp Tissue Kit (QIAGEN®). The primers used for the *lbe* PCR amplification reactions were: 5′-aacgtgctcgagata-acaaatgacc-3′ (forward primer) and 5′-agaagaaccatcgattgctaagaag-3′ (reverse primer). The primers used for the *lbl* PCR amplification reactions were: 5′-atttccgttgatactttggctgag-3′ (forward primer) and 5′-tgttggcgaaatagtgaatatctg-3′ (reverse primer). Methods are as previously described [36]. The sequences of both strands were determined for each line, using 12 overlapping internal primers spaced, on average, 500 nucleotides. At least two independent PCR amplifications were sequenced for each polymorphic site in all *D. melanogaster* strains to prevent possible PCR and sequencing errors. The GenBank accession numbers for the sequences are FJ754496 – FJ754564 and FJ754565 – FJ754633.

### DNA Sequence Analysis

The *lbe* and *lbl* sequences were assembled using the program SeqMan (Lasergene, DNASTAR, Inc.). Multiple alignment was carried out manually and using the program CLUSTAL W [88]. The "sliding window" method of Hudson and Kaplan [83] and most intra-specific analyses were performed using DnaSP v. 4.10.9 [89] and PROSEQ v. 2.9 [90]. Departures from neutral expectations were investigated using HKA [91], Tajima's [35], McDonald and Kreitman's [92], Kelly's [33], and Wall's [34] tests. Simulations based on the coalescent process with or without recombination [93]–[95] were performed with DnaSP and PROSEQ to estimate the probabilities of the observed values of Tajima's *D*, Kelly's *Z_nS_* and Wall's *B* and *Q* statistics and confidence intervals of the nucleotide diversity values. Simulations with 10,000 replicates were conditional on the sample size, the observed number of segregating sites, and the DNA alignment length, with the population recombination rate parameter (*ρ* = 4*N_0_r*) set to the gene estimates. The permutation approach of Hudson et al. [96] was used to estimate the significance of sequence differences between populations and haplotype families and the method of Sawyer [31] to detect gene conversion events. The population recombination rate was analyzed with a permutation-based approach [97].

### Codon-Based Sequence Analyses

Probabilistic Markov codon-substitution models were fitted to coding alignments. Model parameters were estimated using maximum likelihood. These models measure selective pressure using the ratio of nonsynonymous to synonymous substitution rates ω = *d*_N_/*d*_S_, which may vary among sites. Positive or negative selection is evidenced by significant deviations of the ω-ratio from 1. We used models that assume constant synonymous rates M0, M3, M7, M8 [98] and FMutSel0, FMutSel [99] as implemented in PAML v. 4 [100], and a model accounting for variability of synonymous rate over sites GYxHKY Dual GDD 3×3 of [70], later referred as M3-Dual and implemented in HYPHY [69]. Hypotheses concerning selection, codon bias, and rate variability were tested using likelihood ratio tests (LRTs). For a review on the application of codon models see [101]. Models combining coding and noncoding sequences were used to test for positive selection on noncoding regions, as implemented in EvoNC [102]. The strength of selection on noncoding regions was measured by ζ, the ratio of the substitution rate in noncoding regions relative to the synonymous rate in coding regions. Under neutrality, these rates are expected to be similar (ζ≈1). Significant deviations from 1 may be considered as evidence of positive (ζ>1) or negative (ζ<1) selection on noncoding regions. Consequently, the null model allowed two classes of sites in noncoding regions: a neutral class with ζ = 1 and a class of sites evolving under negative selection where the average exonic synonymous rate was higher than the substitution rate in the noncoding regions (ζ<1). The alternative model also allowed two classes of sites, but the rate ratio was estimated for both classes under constraints: ζ≥1 for positive and neutral selection class, and ζ<1 for the negatively selected class. A Bayesian approach was used to predict sites affected by positive selection in both coding and noncoding regions [70], [98], [102], [103].

## Supporting Information

Figure S1 **DNA polymorphism in the** ***lbe*** **gene of 70 strains of** ***Drosophila melanogaster*** **.** Symbols for strains: ER, El Rio; Ven, Venezuela; Bar, Barcelona; letters before and after the number refer to the electrophoretic allele observed in earlier studies at two loci: *esterase-6*, before the hyphen, and *superoxide dismutase*, after the hyphen (S, Slow; F, Fast; US, Ultra Slow). MEL, the *lbe* sequence obtained from GenBank (accession number, NT 033777.2). Lines are arranged successively according to genetic similarity. Numbers on top represent the position of segregating sites and the start of a deletion or insertion. Nucleotides are numbered from the beginning of our sequence. Coding regions of the genes are underlined below the top, reference sequence. Dots indicate same nucleotide as reference sequence. A hyphen represents deleted nucleotides. ▴ denotes a deletion; † absence of a deletion; ▾ insertion; ‡ absence of an insertion. Numbers after symbols for the deletions and insertions refer to the particular deletions and insertions. ▴1, a single nucleotide deletion of G (position 676); ▴2, a single nucleotide deletion of T (position 812); ▴3, a 5-bp deletion of TGGAA (position 1710–1714); ▴4, a 4-bp deletion of TAAA (position 1829–1832); ▴5, a 29-bp deletion of TTCAAATGAAGGTGTTTCGTATAATATCA (position 1876–1904); ▴6, a 30-bp deletion of TCGTATAATATCAATATTCCAACACTACA-A (position 1892–1921); ▾1, a 22-bp insertion of TAGTTGCTCCATGTAACCATGT (position 1953-1974); ▴7, a 8-bp deletion of AGCAACTA (position 1975–1982); ▴8, a 2-bp deletion of AA (position 2006–2007); ▾2, a 10-bp insertion of TGATTTTTTT (position 2008–2017). Coordinates for functional regions of genes are: 1–950 (*lbe*, intron I), 951–1382 (*lbe*, exon II), 1383–2044 (*lbe*, 3′-flanking region).
(DOC)

Figure S2 **DNA polymorphism in the** ***lbl*** **gene of 70 strains of** ***Drosophila melanogaster*** **.** Amino acid replacement polymorphisms are marked with asterisks (nucleotide under selection is in boldface). Coordinates for the functional regions of the gene are: 1–738 (*lbl*, intron I), 738–918 (*lbl*, exon II), 919–1523 (*lbl*, intron II), 1524–1766 (*lbl*, exon III), 1767–2438 (*lbl*, 3′-flanking region). ▾1, a single nucleotide insertion of A (position 973); ▴1, a 78-bp deletion of AATATATTTTTTTGCTGCAAATCTGCTGTTTTTCGCTTTTCTCAGCGAAAT-ATGTACTATTTTCAGTTAAAATATAAT (position 1059–1136); ▾2, a 12-bp insertion of ATAATAAAATAT (position 1132–1143); ▾3, a 24-bp insertion of AAAATATTAATTAATT-TATTATTA (position 1137–1160); ▴2, a 26-bp deletion of CACAAAAGATATCCATTTCTG-GATAT (position 1161–1186); ▴3, a 237-bp deletion of CACAAAAGATATCCATTTCTGG-ATATGAATGAAGTGCATCTTATTCCGACTGACAATTTTGTAGGGAAATGGTAGAGTCGCCTGGCAGTGACTATTTATTTTTCAGTCAACATGTATATAATGTGCATTGTTTTTCCTTTGGCTGAGTAAATGTATCTCGAACTCGACTACAACTCTCTGTTGTTTTTTCTAACCATTTTGTTCTAATGTCAGCAAATTAATAAATATATGCGTCCT (position 1161–1398); ▴4, a single nucleotide deletion of T (position 1263); ▴5, a single nucleotide deletion of A (position 1349); ▴6, a 9-bp deletion of ACACCAGCA (position 1719–1727); ▴7, a 6-bp deletion of GCACCA (1728–1733); ▴8, a 32-bp deletion of CGTTCGCCGTTGAGAATAA-TCGTAAACCATTC (position 2347–2378). Other comments: see Figure S1.
(DOC)

Figure S3 **Fisher exact test of nonrandom associations between pairs of** ***lbe*** **and** ***lbl*** **polymorphisms.** Singleton mutations are excluded from the analysis. Each box in the matrix represents the comparison of two polymorphic sites. Location of the segregating sites on *lbe* and *lbl* genes is shown on the diagonal, which indicates the position of the 5′-flanking, coding, and 3′-flanking regions. 0.01<*P*<0.05 (grey); 0.001<*P*<0.01 (dark grey); *P*<0.001 (black). Intergenic associations are boxed.
(TIF)

Table S1 **Nucleotide diversity and divergence in the** ***lbe*** **gene region of** ***D. melanogaster*** **.**
(DOC)

Table S2 **Nucleotide diversity and divergence in the** ***lbl*** **gene region of** ***D. melanogaster*** **.**
(DOC)

Table S3 **Nucleotide diversity and divergence in the** ***ladybird*** **gene region of** ***D. melanogaster*** **.**
(DOC)

Table S4 **Recombination estimates (** ***ρ*** **).**
(DOC)

Table S5 **Gene conversion events in the** ***lbe*** **and** ***lbl*** **genes of** ***D. melanogaster*** **.**
(DOC)

Table S6 **Linkage disequilibrium between functional regions of the** ***lbe*** **and** ***lbl*** **genes.**
(DOC)

Table S7 **Kelly's (Kelly 1997) and Wall’s (Wall 1999) tests of neutrality for the** ***lbe*** **and** ***lbl*** **gene regions.**
(DOC)

Table S8 **Statistical significance of the binding site cluster for the** ***lbe*** **and** ***lbl*** **genes.**
(DOC)

Text S1 **Nucleotide polymorphism.**
(DOC)

Text S2 **Haplotype structure.**
(DOC)

Text S3 **Linkage disequilibrium.**
(DOC)

Text S4 **Neutrality and maximum likelihood (ML) analysis of selective pressures.**
(DOC)
