# Supplementary material for: Complex Interplay of Evolutionary Forces in the ladybird Homeobox Genes of Drosophila melanogaster
Source: PLoS One. 2011 Jul 22;6(7):e22613. doi: 10.1371/journal.pone.0022613 (PMC3142176; doi:10.1371/journal.pone.0022613)
Supplement: Figure S1 — DNA polymorphism in the lbe gene of 70 strains of Drosophila melanogaster . Symbols for strains: ER, El Rio; Ven, Venezuela; Bar, Barcelona; letters before and after the number refer to the electrophoretic allele observed in earlier studies at two loci: esterase-6, before the hyphen, and superoxide dismutase, after the hyphen (S, Slow; F, Fast; US, Ultra Slow). MEL, the lbe sequence obtained from GenBank (accession number, NT 033777.2). Lines are arranged successively according to genetic similarity. Numbers on top represent the position of segregating sites and the start of a deletion or insertion. Nucleotides are numbered from the beginning of our sequence. Coding regions of the genes are underlined below the top, reference sequence. Dots indicate same nucleotide as reference sequence. A hyphen represents deleted nucleotides. ▴ denotes a deletion; † absence of a deletion; ▾ insertion; ‡ absence of an insertion. Numbers after symbols for the deletions and insertions refer to the particular deletions and insertions. ▴1, a single nucleotide deletion of G (position 676); ▴2, a single nucleotide deletion of T (position 812); ▴3, a 5-bp deletion of TGGAA (position 1710–1714); ▴4, a 4-bp deletion of TAAA (position 1829–1832); ▴5, a 29-bp deletion of TTCAAATGAAGGTGTTTCGTATAATATCA (position 1876–1904); ▴6, a 30-bp deletion of TCGTATAATATCAATATTCCAACACTACA-A (position 1892–1921); ▾1, a 22-bp insertion of TAGTTGCTCCATGTAACCATGT (position 1953-1974); ▴7, a 8-bp deletion of AGCAACTA (position 1975–1982); ▴8, a 2-bp deletion of AA (position 2006–2007); ▾2, a 10-bp insertion of TGATTTTTTT (position 2008–2017). Coordinates for functional regions of genes are: 1–950 (lbe, intron I), 951–1382 (lbe, exon II), 1383–2044 (lbe, 3′-flanking region). (DOC) [file pone.0022613.s001.doc]

-------------------------------------------------------------------------------------------------------------------------------------------------------

11111111111111111 111 11111111 1 111 1 1112 2 22

11223445555666 68 8999900001223555555667 788 88888888 8 999 9 9990 0 00

2325554272677677 91 4112245582890011234161 412 34556777 9 145 7 8880 0 34

2116028592616936 72 4462805881927078602780 549 61049126 2 273 5 3796 8 74

ER-F-357F CGACACTACCGCCAA† C† CTTTTTGCCCGCCCTTAGCAT† CT† AGCCGAG† † AT‡ † ATT† ‡ TC

**Group-1**

ER-F-611F ................ .. ...................... ... ........ . ... . .... . ..

ER-S-510S ................ .. ...................... ... ........ . ... . .... . ..

ER-S-549S ................ .. ...................... ... ........ . ... . .... . ..

ER-S-2588S ................ .. ...................... ... ........ . ... . .... . ..

ER-S-255S ................ .▲2...................... ... ........ . ... . .... . ..

ER-F-96S .A..G........... .. ...................... ... ........ . ... . .... . ..

Ven-S-2F ................ .. ...................... ... ........ . ... . .... . ..

Ven-S-3F ................ .. ...................... ... ........ . ... . .... . ..

Ven-S-8F ................ .. ...................... ... ........ . ... . .... . ..

Ven-S-11F ................ .. ...................... ... ........ . ... . .... . ..

Bar-S-78F ................ .. ...................... ... ........ . ... . .... . ..

Bar-S-99F .......G........ .. ...................... ... ........ . ... . .... . ..

Bar-F-79F ................ .. ...................... ... ........ . ..▼1▲7.... . ..

Bar-S-44F ................ .. ...................... ... ........ . ..▼1▲7.... . ..

Ven-S-4F ................ .. ...................... ... ........ . ..▼1▲7.... . ..

Ven-S-15F ................ .. ...................... ... ........ . ..▼1▲7.... . ..

Ven-S-16F ................ .. ...................... ... ........ . ..▼1▲7.... . ..

Ven-S-20F ................ .. ...................... ... ........ . ..▼1▲7.... . ..

ER-F-517F ................ .. ...................... ... ........ . ..▼1▲7.... . ..

ER-F-517S ................ .. ...................... ... ........ . ..▼1▲7.... . ..

ER-F-1461S ................ .. ...................... ... ........ . ..▼1▲7.... . ..

ER-S-174F ................ .. ...................... ... ........ . ..▼1▲7.... . ..

ER-S-501F ................ .. ...................... ... ........ . ..▼1▲7.... . ..

ER-S-521F ................ .. ...................... ... ........ . ..▼1▲7.... . ..

ER-S-1224F ................ .. ...................... ... ........ . ..▼1▲7.... . ..

ER-S-377F ................ .. ...................... T.. ........ . ..▼1▲7.... . ..

ER-F-775F ................ .. .....................▲3... ........ . ..▼1▲7.... . ..

ER-S-26F .A...T.......... .. ...................... ... ........ . ..▼1▲7.... . ..

**Sub-group-1a**

ER-S-521S .A....CG........ .. ..............C.C..CC. ... ........ . ... . .... . ..

ER-S-581F .A....CG........ .. ..............C.C..CC. ... ........ . ... . .... . ..

Ven-S-5F .A....CG........ .. ..............C.C..CC. ... ........ . ... . .... . ..

Ven-S-7F .A....CG........ .. ..............C.C..CC. ... ........ . ... . .... . ..

Ven-S-12F .A....CG........ .. ..............C.C..CC. ... ........ . ... . .... . ..

Ven-S-13F .A....CG........ .. ..............C.C..CC. ... ........ . ... . .... . ..

Ven-S-14F .A....CG........ .. ..............C.C..CC. ... ........ . ... . .... . ..

Ven-S-17F .A....CG........ .. ..............C.C..CC. ... ........ . ... . .... . ..

Ven-S-18F .A....CG........ .. ..............C.C..CC. ... ........ . ... . .... . ..

Ven-S-22F .A....CG........ .. ..............C.C..CC. ... ........ . ... . .... . ..

Ven-S-23F .A....CG........ .. ..............C.C..CC. ... ........ . ... . .... . ..

**Sub-group-2a**

ER-S-501S ......CG........ A. T.AA.C..TG.T.TCGC...C. ... G...A... . C.. . .A.▲8▼2..

Bar-S-86F ......CG........ A. T.AA.C..TG.T.TCGC...C. ... G...A... . C.. . .A.▲8▼2..

Bar-F-96S ......CG........ A. T.AA.C..TG.T.TCGC...C. ... G...A... . C.. . .A.▲8▼2..

ER-US-255F .......GAT....C. .. T..A.CAT.GATG.CGC..... ... G...A... . C.. . .A.▲8▼2..

Bar-F-93F .......GAT....C. .. T..A.CAT.G.T..CGC..... ... G...A... . C.. . .A.▲8▼2..

Zim-F-S18 ......CG.......▲1.. T..A.CAT.GATG.CGC..... ... G...A... . CC. . .A.▲8▼2..

**Group-2**

Ven-S-21F ......CG.......▲1.. T..A.C..TG..G.C.CC.... ..▲4G.TT.T.. ▲6-.. . .AA. . .T

Ven-S-10F ......CG.......▲1.. T..A.C..TG.TG.C.CC.... ..▲4G.TT.T.. ▲6-.. . .AA. . .T

Bar-F-77F ......CG.......▲1.. T..A.C..TG.TG.C.CC.... ..▲4G.TT.T.. ▲6-.. . .AA. . .T

Bar-S-158F ......CG.......▲1.. T..A.C..TG.TG.C.CC.... ..▲4G.TT.T.. ▲6-.. . .AA. . .T

ER-S-438S ......CG.......▲1.. T..A.C..TG.TG.C.CC.... ..▲4G.TT.T.. ▲6-.. . .AA. . .T

ER-S-565F ......CG.......▲1.. T..A.C..TG.TG.C.CC.... ..▲4G.TT.T.. ▲6-.. . .AA. . .T

Bar-S-19F ....T.CG.......▲1.. T..A.C..TG.TG.C.CC.... ..▲4G.TT.T.. ▲6-.. . .AA. . .T

Bar-S-80F ....T.CG.......▲1.. T..A.C..TG.TG.C.CC.... ..▲4G.TT.T.. ▲6-.. . .AA. . .T

Bar-S-95F ....T.CG.......▲1.. T..A.C..TG.TG.C.CC.... ..▲4G.TT.T.. ▲6-.. . .AA. . .T

ER-S-483F T.....CG.......▲1.. T..A.C..TG.TG.C.CC.... ..▲4G.TT.T.. ▲6-.. . .AA. . .T

ER-S-968F T.....CG.......▲1.. T..A.C..TG.TG.C.CC.... ..▲4G.TT.T.. ▲6-.. . .AA. . .T

Zim-S-44F ......CG.TAT...▲1.. TC.ACC..TG.T..C.C.T... ..▲4G.TT.T.. ▲6-.. . .AA. . .T

**Recombinants:**

ER-S-114S ................ .. ...................... ..▲4G.TT.T.. ▲6-.. . .AA. . .T

ER-F-531F ................ .. ...A.C..TG.TG.C.CC.... ... ........ . ... . G... . ..

ER-F-274F ......CG........ .. ...................... ... ........ . ..▼1▲7.... . ..

Bar-S-60F ......CG.......▲1.. T..A.C..TG.TG.C.CC.... ... ........ . ..▼1▲7.... . ..

Bar-S-48F .......GA....T.▲1.. T..A.C..TG.TG.C.CC.... .A. GT....A. . ... . .A.. . A.

Bar-44S ..G............. .. T.AA.C..TG.T.......... ... .......▲5. ..▼1▲7.... . ..

Bar-S-24F ................ .. ..............C.C..CC. ... ........ . ... . .... . ..

Bar-S-89F .A....CG........ .. ...................... ... ........ . ... . .... . ..

Bar-F-7F .A....CG........ .. ..AA.................. ... ........ . ... . .... . ..

Bar-S-119F ...A............ .. .....C..TG............ ... ........ . ..▼1▲7.... . ..

Zim-S-S34 .......G.TATT...... ...A..........C.CC.... ... GT....A. . ... . .A.. . A.

MEL .......GAT....C.... T..A.CAT.GATG......... ... ........ . ... . .... . ..

-------------------------------------------------------------------------------------------------------------------------------------------------------

Figure S1.
