# Supplementary material for: Complex Interplay of Evolutionary Forces in the ladybird Homeobox Genes of Drosophila melanogaster
Source: PLoS One. 2011 Jul 22;6(7):e22613. doi: 10.1371/journal.pone.0022613 (PMC3142176; doi:10.1371/journal.pone.0022613)
Supplement: Figure S2 — DNA polymorphism in the lbl gene of 70 strains of Drosophila melanogaster . Amino acid replacement polymorphisms are marked with asterisks (nucleotide under selection is in boldface). Coordinates for the functional regions of the gene are: 1–738 (lbl, intron I), 738–918 (lbl, exon II), 919–1523 (lbl, intron II), 1524–1766 (lbl, exon III), 1767–2438 (lbl, 3′-flanking region). ▾1, a single nucleotide insertion of A (position 973); ▴1, a 78-bp deletion of AATATATTTTTTTGCTGCAAATCTGCTGTTTTTCGCTTTTCTCAGCGAAAT-ATGTACTATTTTCAGTTAAAATATAAT (position 1059–1136); ▾2, a 12-bp insertion of ATAATAAAATAT (position 1132–1143); ▾3, a 24-bp insertion of AAAATATTAATTAATT-TATTATTA (position 1137–1160); ▴2, a 26-bp deletion of CACAAAAGATATCCATTTCTG-GATAT (position 1161–1186); ▴3, a 237-bp deletion of CACAAAAGATATCCATTTCTGG-ATATGAATGAAGTGCATCTTATTCCGACTGACAATTTTGTAGGGAAATGGTAGAGTCGCCTGGCAGTGACTATTTATTTTTCAGTCAACATGTATATAATGTGCATTGTTTTTCCTTTGGCTGAGTAAATGTATCTCGAACTCGACTACAACTCTCTGTTGTTTTTTCTAACCATTTTGTTCTAATGTCAGCAAATTAATAAATATATGCGTCCT (position 1161–1398); ▴4, a single nucleotide deletion of T (position 1263); ▴5, a single nucleotide deletion of A (position 1349); ▴6, a 9-bp deletion of ACACCAGCA (position 1719–1727); ▴7, a 6-bp deletion of GCACCA (1728–1733); ▴8, a 32-bp deletion of CGTTCGCCGTTGAGAATAA-TCGTAAACCATTC (position 2347–2378). Other comments: see Figure S1. (DOC) [file pone.0022613.s002.doc]

--------------------------------------------------------------------------------------------------------------------------------------------------------------------------------------------------------------------------------------

1111111111111 11 1 111 1 11111111 1111111 111111111111111111 1 1111 111122222222222222222 2222

1113333345566789999 0000000000000 01 1 111 1 11122222 2233333 333344444444456777 7 7888 899900000111222233333 3344

1343483344503949230267 0001122333455 83 3 446 6 67704556 7801234 566802234468909011 2 3027 813923358138333501144 5911

1076432835194908373083 5896729039929 52 7 351 1 83471273 3727309 413292350538767829 8 5025 857450174253249980577 8613

ER-F-357F CGGCAAATTGGCCAGTAGCTA‡ GTTATTAGTGGC† T‡ ‡ --† † GCCCCCT† TAGCCA▲5ATTAGGCTCAGCGA**AC**A† ▲7**T**GCA CAACCGGCGATTGAATATC-▲8-AAT

** *

**Group-1**

ER-F-517S ...................... ............. .. . --. . ........ ....... .................. . .... ...................-. -...

ER-F-517F ...................... ............. .. . --. . ........ ....... .................. . .... ...................-. -...

ER-S-114S ...................... ............. .. . --. . ........ ....... .................. . .... ...................-. -...

ER-S-2588S ...................... ............. .. . --. . ........ ....... .................. . .... ...................-. -...

Bar-S-119F ...................... ............. .. . --. .......... ....A.. .................. . .... ...................-. -...

ER-S-549S ...........G.......... ............. .. . --. . ........ ....... .................. . .... ...................-. -..G

ER-S-581F ...........G.......... ............. .. . --. . ........ ....... .................. . .... ...................-. -..G

Ven-S-13F ?..........G.......... ............. .. . --. . ........ ....... .................. . .... ...................-. -..G

Ven-S-7F ?..........G.......... ............. .. . --. . ........ ....... .................. . .... ...................-. -..G

Ven-S-5F ?..........G.......... ............. .. . --. . ........ ....... .................. . .... ...................-. -..G

Ven-S-12F ?..........G.......... ............. .. . --. . ........ ....... .................. . .... ...................-. -..G

Ven-S-22F ?..........G.......... ............. .. . --. . ........ ....... .................. . .... ...................-. -..G

Ven-S-18F ?..........G.......... ............. .. . --. . ........ ....... .................. . .... ...................-. -..G

Ven-S-23F ?..........G.......... ............. .. . --. . ........ ....... .................. . .... ...................-. -..G

Ven-S-17F ?..........G.......... ............. .. . --. . ........ ....... .................. . .... ...................-. -..G

Ven-S-14F ?..........G.......... ............. .. . --. . ........ ....... .................. . .... ...................-. -..G

ER-S-1224F ...........G.......... ............. .. . --. . ........ ...A... .................. . .... ...................-. -...

ER-S-521S ...........G....G..... ............. .. . --. . ........ ....... .................. . .... ....T..............-. -...

ER-S-501F ...T.......G.......... ............. .. . --. . ........ ...A... .................. . .... ...................-. -...

Bar-F-79F ...........GT...G..... ............. .. . --. . ........ ....... .................. . .... ...................-. -...

Ven-S-15F ?..........G...GG..... ............. .. . --. . ........ ....... .................. . .... A.......TTG.....GG.C† G..G

Ven-S-4F ?..........G...GG..... ............. .. . --. . ........ ....... .....C............ . .... A.......TTG.....GG.C† G..G

Ven-S-16F ?...G......G...GG..... ............. .. . --. . ........ ....... .....C............ . .... A.......TTG.....GG.C† G..G

**Sub-group-1a**

Bar-F-7F ?....T.CGCAGT...G..... .......A..A.. .▼2. T-▲2. ---..T.. .....C† G.CG.............. . .A.. ...A..TTT.G...TCGG.C† G..G

Bar-S-86F .....T.CGCAGT...G..... .......A..A.. .▼2. T-▲2. ---..T.. .....C† G.CG.............. . .A.. ...A..T.T.G...TCGG.C† G..G

Ven-S-11F ?....T.CGCAGT...G..... .......A..A.. .▼2. T-▲2. ---..T.. .....C† G.CG.............. . .A.. ...A..T.T.G...TCGG.C† G..G

Ven-S-2F ?....T.CGCAGT...G..... .......A..A.. .▼2. T-▲2. ---..T.. .....C† G.CG.............. . .A.. ...A..T.T.G...TCGG.C† G..G

Ven-S-8F ?....T.CGCAGT...G..... .......A..A.. .▼2. T-▲2. ---..T.. .....C† G.CG.............. . .A.. ...A..T.T.G...TCGG.C† G..G

Ven-S-3F ?....T.CGCAGT...G..... .......A..A.. .▼2. T-▲2. ---..T.. .....C† G.CG.............. . .A.. ...A..T.T.G...TCGG.C† G..G

ER-F-611F .....T.CGCAGT...G..... .......A..A.. .▼2. T-▲2. ---..T.. .....C† G.CG.............. . .A.. ...A..T.T.G...TCGG.C† G..G

**Sub-group-1b**

ER-S-26F TAT..T.CGC.G.T..G.T... .........C.G. A. . --. . TGA.G.C. ....AC† G.CG....GTAT.CC.C▲6† CA.. ...................C† G..G

ER-S-565F TAT..T.CGC.G.T..G.T... .........C.G. A. . --. . TGA.G.C. ....AC† G.CG....GTAT.CC.C▲6† CA.. ...................C† G..G

ER-S-483F ...........GT...G.T... .........C.G. A. . --. . ..A.G... ....AC† G.CG....GTAT.CC.C▲6† C... ...................-. -...

ER-S-968F ...........GT...G.T... .........C.G. A. . --. . ..A.G... ....AC† G.CG....GTAT.CC.C▲6† C... ...................-. -...

ER-F-775F .....T.....GT...G.T... .........C.G. A. . --. . ..A.G... ....AC† G.CG....GTAT.CC.C▲6† C... ...................-. -...

ER-S-174F .....T.....GT...G.T... .........C.G. A. . --. . ..A.G... ....AC† G.CG....GTAT.CC.C▲6† CA.. ..T................-. -...

Bar-S-158F .....T.....GT...G.T... .........C.G. A. . --. ...A.G.... ....AC† G.CG....GTAT.CC.C▲6† C... ...................-. -...

**Group-2**

Bar-44S ...........G....G...C. ....A.G.....▲1-. ▼3TA. ▲3------.. ------- ----TCT.GTA.A..T.. † .... ...................-. -...

Bar-S-80F ?..........G....G...C. ....A.G.....▲1-. ▼3TA. ▲3------.. ------- ----TCT.GTA.A..T.. † .... ...................-. -...

MEL ...........G....G...C. ....A.G.....▲1-. ▼3TA. ▲3------.. ------- ----TCT.GTA.A..T.. . .... ...................-. -...

Bar-S-60F ?...............G...C. ....A.G.....▲1-. ▼3TA. ▲3------.. ------- ----TCT.GTA.A..... . .... ...................-. -...

Bar-F-77F .....T..........G...C. ....A.G.....▲1-. ▼3TA. ▲3------.. ------- ----TCT.GTA.A..T.. . .... ...................-. -...

ER-S-377F .....TCCGC......G...C. ....A.G.....▲1-. ▼3TA. ▲3------.. ------- ----TCT.GTA.A..... . .A.. A..................-. -...

ER-F-274F .....TCCGC......G...C. ....A.G.....▲1-. ▼3TA. ▲3------.. ------- ----TCT.GTA.A..T.. . .... ...................-. -...

Bar-F-96S ?....TCCGC......G...C. ....A.G.....▲1-. ▼3TA. ▲3------.. ------- ----TCT.GTA.A..T.. . .... ...................-. -...

Bar-S-89F .....T.CGC......G...C. ....A.G.....▲1-. ▼3TA. ▲3------.. ------- ----TCT.GTA.A..T.. † .... ...................-. -...

ER-S-501S .....T.CGC......G...C. ....A.G.A...▲1-. ▼3TA. ▲3------.. ------- ----TCT.GTA.A..T.. . .... ...................-. -...

ER-US-255F .....T.CGC......G...C. ....A.G.A...▲1-. ▼3TA. ▲3------.. ------- ----TCT.GTA.A..T.. . .... ...................-. -...

Bar-S-19F ?....T.CGC......G...C. ....A.G.....▲1-. ▼3TA. ▲3------.. ------- ----TCT.GTA.A..T.. . .... ...................-. -...

Bar-S-95F .....T.CGC......G...C. ....A.G.....▲1-. ▼3TA. ▲3------.. ------- ----TCT.GTA.A..T.. . .... ...................-. -...

**Recombinants:**

ER-S-438S ...........G.......... .......A..A.. .▼2. T-▲2. ---..T.. .....C† G.CG.............. . .... ...................-. -...

ER-S-255S ...........G.......... .......A..A.. .▼2. T-▲2. ---..T.. .....C† G.CG....GTA..C.... . .... ...................-. -...

ER-F-1461S .....TCCGC............ .......A..A.. .▼2. T-▲2. ---..T.. .....C† G.CG....GTA..C.... . .... ...................-. -...

Bar-S-24F ?..................... .......A..A.. .▼2. T-▲2. ---..T.. .....C† G.CG.............. . .... A..................-. -..G

Bar-S-44F ?...................C. .......A..A.. .▼2. T-▲2. ---..T.. ....... .................. . .... ...................-. -...

ER-F-531F ...........G.......... ............. .. . --. . ........ ..T.... .............C.... . .A.. ...A..T.T.G...TCGG.C† G..G

ER-S-521F ...........G.......... ............. .. . --. . ........ ..T.... .............C.... . .A.. ...A..T.T.G...TCGG.C† G..G

ER-F-96S .......CGC.G.......G.. ............. .. . --. . ........ ....... .................. . .... ...A..T.T.G...TCGG.C† G..G

Bar-S-99F .....T.CGC......G...C. ............. .. . --. .......... ....... .................. . .... ...................-. -...

Bar-S-78F .....T.CGC......G...C. ....A.G...... .. . --. ...A...... ....... .................. . .... ...................-. -...

Ven-S-10F ?....T.CGCAGT...G..... ............. .. . --. . ........ ....... .................. † CA.. ......T............C† G..G

Ven-S-21F ?....T.CGCAGT...G..... ............. .. . --. . ........ ....... .................. † CA.. ......T............C† G..G

Zim-S-S34 .....T.CGC.G....G.T... ..C......C.G. A. . --. . ..A....▲4CG.... .....C..........C▲6† CA.. A.......T.G.C.T.GGTC† G..G

Bar-S-48F ?....T.....G..A.G..... ...T..G.....▲1-. ▼3TA. ▲3------.. ------- ----TC........C.C▲6† CATC .T......T.G...T.GG.-. -...

Bar-F-93F .....T.CGC.G....G...C. ....A.G.....▲1-. ▼3AC. ▲3------.. ------- ----TCT.GTA..C.T.. † .A.. .....T..T.GAC.T.GGTC† G..G

Ven-S-20F ?....T.....G....G..... ...T..G.....▲1-. ▼3TA. ▲3------.. ------- ----TC............ . .... A.......TTG.....GG.C† G..G

Zim-F-S18 T....T.C...G.......... .C.T..G.....▲1-. ▼3TA. ▲3------.. ------- ----TCTAGTA....... † .A.. ........T.G...T.GG.-. -.C.

Zim-S-44F .....T.....G.........▼1............. .. . --. . ..AA.... .....C† GGCG....GTAT.C.... † .A.. A.......T.G..TTC...A† AT..

ER-S-510S ...........G....GA.... C....C....... A. . --. . ..A.G... ....AC† G.CG....GTAT.C.... † CA.. ......T............C† G..G

--------------------------------------------------------------------------------------------------------------------------------------------------------------------------------------------------------------------------------------

Figure S2.
