# Supplementary figures and images for: Complex Interplay of Evolutionary Forces in the ladybird Homeobox Genes of Drosophila melanogaster
Source: PLoS One. 2011 Jul 22;6(7):e22613. doi: 10.1371/journal.pone.0022613 (PMC3142176; doi:10.1371/journal.pone.0022613)

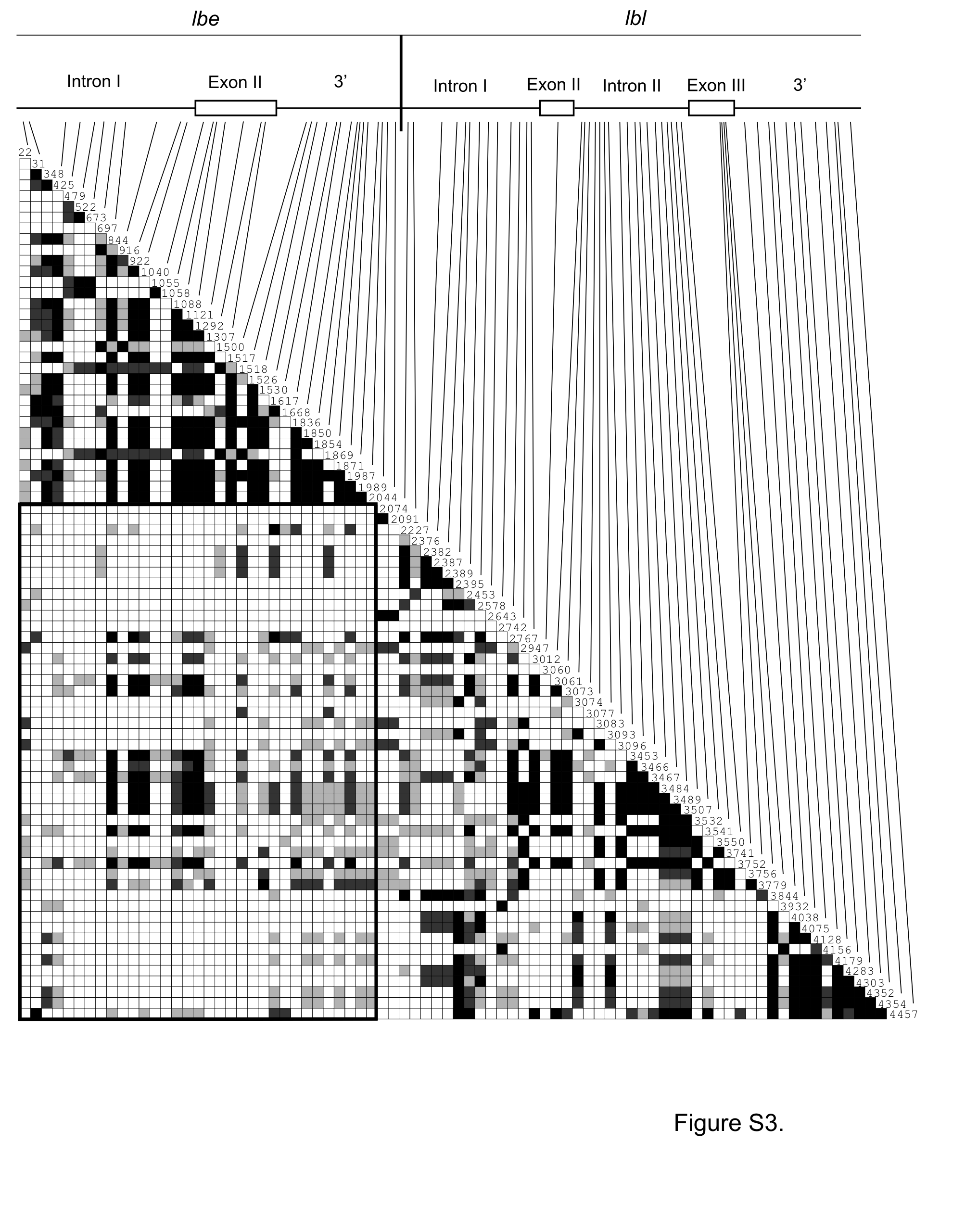

Supplement: Figure S3 — Fisher exact test of nonrandom associations between pairs of lbe and lbl polymorphisms. Singleton mutations are excluded from the analysis. Each box in the matrix represents the comparison of two polymorphic sites. Location of the segregating sites on lbe and lbl genes is shown on the diagonal, which indicates the position of the 5′-flanking, coding, and 3′-flanking regions. 0.01<P<0.05 (grey); 0.001<P<0.01 (dark grey); P<0.001 (black). Intergenic associations are boxed. (TIF) [file pone.0022613.s003.tif]
