# Supplementary material for: Complex Interplay of Evolutionary Forces in the ladybird Homeobox Genes of Drosophila melanogaster
Source: PLoS One. 2011 Jul 22;6(7):e22613. doi: 10.1371/journal.pone.0022613 (PMC3142176; doi:10.1371/journal.pone.0022613)
Supplement: Table S1 — Nucleotide diversity and divergence in the lbe gene region of D. melanogaster . (DOC) [file pone.0022613.s004.doc]

**Table S1.** Nucleotide diversity and divergence in the *lbe* gene region of *D. melanogaster*

|  |  | *lbe* exon II | | |  |  | Full sequence | |
| --- | --- | --- | --- | --- | --- | --- | --- | --- |
|  | Intron I | Syn | Nsyn | Total | 3’-fl. region | Ncod | Silent | All sites |
| N | 948 | 100 | 329 | 429 | 568 | 1516 | 1616 | 1945 |
| All, 70 lines |  |  |  |  |  |  |  |  |
| S | 21 (7) | 8 (0) | 0 (0) | 8 (0) | 23 (5) | 44 (12) | 52 (12) | 52 (12) |
| π | 0.0035 | 0.0244 | 0 | 0.0057 | 0.0090 | 0.0055 | 0.0067 | 0.0056 |
| θ | 0.0046 | 0.0166 | 0 | 0.0039 | 0.0084 | 0.0060 | 0.0068 | 0.0056 |
| *K_mel-sim_* | 0.0257 | 0.1425 | 0 | 0.0309 | 0.0632 | 0.0393 | 0.0454 | 0.0374 |
| *K_mel-sec_* | 0.0330 | 0.1425 | 0 | 0.0309 | 0.0705 | 0.0469 | 0.0526 | 0.0433 |
| *K_mel-yak_* | 0.0792 | 0.1791 | 0 | 0.0382 | 0.1333 | 0.0986 | 0.1035 | 0.0845 |
| Barcelona,19 lines |  |  |  |  |  |  |  |  |
| S | 14 (5) | 7 (2) | 0 (0) | 7 (2) | 19 (5) | 33 (10) | 40 (12) | 40 (12) |
| π | 0.0039 | 0.0271 | 0 | 0.0063 | 0.0108 | 0.0065 | 0.0078 | 0.0065 |
| θ | 0.0042 | 0.0200 | 0 | 0.0047 | 0.0095 | 0.0062 | 0.0071 | 0.0059 |

**Table S1 (continued).**

|  |  | *lbe* exon II | | |  |  | Full sequence | |
| --- | --- | --- | --- | --- | --- | --- | --- | --- |
|  | Intron I | Syn | Nsyn | Total | 3’-fl. region | Ncod | Silent | All sites |
| N | 948 | 100 | 329 | 429 | 568 | 1516 | 1616 | 1945 |
| El Rio, 28 lines |  |  |  |  |  |  |  |  |
| S | 13 (7) | 8 (3) | 0 (0) | 8 (3) | 17 (3) | 30 (10) | 38 (13) | 38 (13) |
| π | 0.0026 | 0.0208 | 0 | 0.0049 | 0.0074 | 0.0045 | 0.0055 | 0.0046 |
| θ | 0.0035 | 0.0205 | 0 | 0.0048 | 0.0075 | 0.0050 | 0.0060 | 0.0050 |
| Venezuela, 19 lines |  |  |  |  |  |  |  |  |
| S | 5 (0) | 5 (1) | 0 (0) | 5 (1) | 12 (0) | 17 (0) | 22 (1) | 22 (1) |
| π | 0.0021 | 0.0090 | 0 | 0.0021 | 0.0062 | 0.0037 | 0.0040 | 0.0033 |
| θ | 0.0015 | 0.0143 | 0 | 0.0033 | 0.0058 | 0.0032 | 0.0038 | 0.0032 |

**Table S1 (continued).**

Calculations based on 70 *D. melanogaster* lines derived from three populations: Barcelona, El Rio (California) and Venezuela, plus three lines from Zimbabwe and one *lbe* sequence from GenBank (accession number NT_033777.2). N, number of sites (indels excluded); S, polymorphic sites (number of singletons in parentheses); π, average number of nucleotide differences per site among all pairs of sequences [104, p. 256]; θ, average number of segregating nucleotide sites among all sequences, based on the expected distribution of neutral variants in a panmictic population at equilibrium [105]; *K_mel-sim_*, *K_mel-sec_*, and *K_mel-yak_* refer to the nucleotide differences between *D. melanogaster* and *D. simulans*, *D. sechellia* or *D. yakuba*, respectively; Syn, synonymous sites; Nsyn, nonsynonymous sites; Ncod, noncoding (intronic and flanking) regions; Silent, silent sites (synonymous and noncoding sites).
