# Supplementary material for: Complex Interplay of Evolutionary Forces in the ladybird Homeobox Genes of Drosophila melanogaster
Source: PLoS One. 2011 Jul 22;6(7):e22613. doi: 10.1371/journal.pone.0022613 (PMC3142176; doi:10.1371/journal.pone.0022613)
Supplement: Table S2 — Nucleotide diversity and divergence in the lbl gene region of D. melanogaster . (DOC) [file pone.0022613.s005.doc]

**Table S2.** Nucleotide diversity and divergence in the *lbl* gene region of *D. melanogaster*

|  |  | *lbl* exon II + exon III | | |  |  |  | Full sequence | |
| --- | --- | --- | --- | --- | --- | --- | --- | --- | --- |
|  | Intron I | Syn | Nsyn | Total | Intron II | 3’-fl. region | Ncod | Silent | All sites |
| N | 725 | 94 | 311 | 405 | 264 | 643 | 1632 | 1726 | 2037 |
| All, 70 lines |  |  |  |  |  |  |  |  |  |
| S | 16 (3) | 3 (1) | 3 (0) | 6 (1) | 24 (6) | 25 (11) | 65 (20) | 68 (21) | 71 (21) |
| π | 0.0053 | 0.0049 | 0.0025 | 0.0031 | 0.0220 | 0.0069 | 0.0086 | 0.0084 | 0.0075 |
| θ | 0.0046 | 0.0066 | 0.0020 | 0.0031 | 0.0189 | 0.0081 | 0.0083 | 0.0082 | 0.0072 |
| *K_mel-sim_* | 0.0357 | 0.0585 | 0.0099 | 0.0209 | 0.1200 | 0.0674 | 0.0599 | 0.0598 | 0.0517 |
| *K_mel-sec_* | 0.0299 | 0.0760 | 0.0112 | 0.0257 | 0.1223 | 0.0781 | 0.0623 | 0.0631 | 0.0547 |
| *K_mel-yak_* | 0.0736 | 0.1496 | 0.0140 | 0.0452 | 0.2772 | 0.1885 | 0.1411 | 0.1416 | 0.1186 |
| Barcelona, 19 lines |  |  |  |  |  |  |  |  |  |
| S | 10 (2) | 2 (1) | 3 (0) | 5 (1) | 7 (4) | 19 (9) | 46 (15) | 48 (16) | 51 (16) |
| π | 0.0050 | 0.0032 | 0.0029 | 0.0030 | 0.0247 | 0.0062 | 0.0087 | 0.0084 | 0.0075 |
| θ | 0.0040 | 0.0061 | 0.0028 | 0.0035 | 0.0184 | 0.0085 | 0.0081 | 0.0080 | 0.0072 |

**Table S2 (continued).**

|  |  | *lbl* exon II + exon III | | |  |  |  | Full sequence | |
| --- | --- | --- | --- | --- | --- | --- | --- | --- | --- |
|  | Intron I | Syn | Nsyn | Total | Intron II | 3’-fl. region | Ncod | Silent | All sites |
| N | 725 | 94 | 311 | 405 | 264 | 643 | 1632 | 1726 | 2037 |
| El Rio, 28 lines |  |  |  |  |  |  |  |  |  |
| S | 14 (2) | 3 (1) | 3 (0) | 6 (1) | 20 (3) | 13 (3) | 47 (8) | 50 (9) | 53 (9) |
| π | 0.0054 | 0.0082 | 0.0030 | 0.0042 | 0.0219 | 0.0049 | 0.0079 | 0.0079 | 0.0072 |
| θ | 0.0049 | 0.0082 | 0.0025 | 0.0038 | 0.0195 | 0.0052 | 0.0073 | 0.0074 | 0.0066 |
| Venezuela, 19 lines |  |  |  |  |  |  |  |  |  |
| S | 9 (1) | 0 (0) | 1 (0) | 1 (0) | 6 (3) | 11 (0) | 26 (4) | 26 (4) | 27 (4) |
| π | 0.0051 | 0 | 0.0006 | 0.0005 | 0.0049 | 0.0074 | 0.0060 | 0.0056 | 0.0048 |
| θ | 0.0036 | 0 | 0.0009 | 0.0007 | 0.0065 | 0.0049 | 0.0046 | 0.0043 | 0.0038 |

For comments see Table S1.
