# Supplementary material for: Complex Interplay of Evolutionary Forces in the ladybird Homeobox Genes of Drosophila melanogaster
Source: PLoS One. 2011 Jul 22;6(7):e22613. doi: 10.1371/journal.pone.0022613 (PMC3142176; doi:10.1371/journal.pone.0022613)
Supplement: Table S3 — Nucleotide diversity and divergence in the ladybird gene region of D. melanogaster . (DOC) [file pone.0022613.s006.doc]

***Table S3.*** *Nucleotide diversity and divergence in the ladybird gene region* of D. melanogaster*

|  | Syn | Nsyn | Cod | Ncod | Silent | Total |
| --- | --- | --- | --- | --- | --- | --- |
| BAR |  |  |  |  |  |  |
| N | 194 | 640 | 834 | 3154 | 3348 | 3988 |
| S | 9 (3) | 3 (0) | 12 (3) | 79 (25) | 88 (28) | 91 (28) |
| π | 0.0156 | 0.0014 | 0.0047 | 0.0076 | 0.0081 | 0.0070 |
| θ | 0.0133 | 0.0013 | 0.0041 | 0.0072 | 0.0075 | 0.0065 |
| *K_mel-sim_* | 0.1040 | 0.0053 | 0.0271 | 0.0493 | 0.0525 | 0.0445 |
| *K_mel-sec_* | 0.1130 | 0.0059 | 0.0295 | 0.0546 | 0.0579 | 0.0492 |
| *K_mel-yak_* | 0.1689 | 0.0071 | 0.0426 | 0.1187 | 0.1217 | 0.1009 |
| ER |  |  |  |  |  |  |
| N | 194 | 640 | 834 | 3177 | 3371 | 4011 |
| S | 11 (4) | 3 (0) | 14 (4) | 77 (18) | 88 (22) | 91 (22) |
| π | 0.0147 | 0.0015 | 0.0045 | 0.0062 | 0.0067 | 0.0058 |
| θ | 0.0146 | 0.0012 | 0.0043 | 0.0062 | 0.0067 | 0.0058 |
| *K_mel-sim_* | 0.1007 | 0.0047 | 0.0259 | 0.0500 | 0.0529 | 0.0448 |
| *K_mel-sec_* | 0.1096 | 0.0051 | 0.0281 | 0.0552 | 0.0583 | 0.0494 |
| *K_mel-yak_* | 0.1649 | 0.0067 | 0.0416 | 0.1202 | 0.1229 | 0.1020 |

**Table S3 (continued).**

|  | Syn | Nsyn | Cod | Ncod | Silent | Total |
| --- | --- | --- | --- | --- | --- | --- |
| VEN |  |  |  |  |  |  |
| N | 197 | 646 | 843 | 3172 | 3369 | 4015 |
| S | 5 (1) | 1 (0) | 6 (1) | 43 (4) | 48 (5) | 49 (5) |
| π | 0.0046 | 0.0003 | 0.0013 | 0.0048 | 0.0048 | 0.0041 |
| θ | 0.0073 | 0.0004 | 0.0020 | 0.0039 | 0.0041 | 0.0035 |
| *K_mel-sim_* | 0.0931 | 0.0061 | 0.0255 | 0.0495 | 0.0521 | 0.0443 |
| *K_mel-sec_* | 0.1018 | 0.0053 | 0.0267 | 0.0548 | 0.0575 | 0.0487 |
| *K_mel-yak_* | 0.1585 | 0.0063 | 0.0400 | 0.1199 | 0.1222 | 0.1013 |
| Total** |  |  |  |  |  |  |
| N | 194 | 640 | 834 | 3148 | 3342 | 3982 |
| S | 11 (1) | 3 (0) | 14 (1) | 109 (32) | 120 (33) | 123 (33) |
| π | 0.0150 | 0.0012 | 0.0044 | 0.0071 | 0.0076 | 0.0066 |
| θ | 0.0118 | 0.0010 | 0.0035 | 0.0072 | 0.0075 | 0.0064 |
| *K_mel-sim_* | 0.1007 | 0.0048 | 0.0260 | 0.0497 | 0.0527 | 0.0446 |
| *K_mel-sec_* | 0.1096 | 0.0054 | 0.0284 | 0.0548 | 0.0580 | 0.0491 |
| *K_mel-yak_* | 0.1649 | 0.0067 | 0.0416 | 0.1189 | 0.1217 | 0.1009 |

*The *ladybird* region includes *lbe* and *lbl* genes. **Calculations are based on 70 *D. melanogaster* lines from three population (Barcelona, BAR; El Rio, ER; and Venezuela, VEN) plus three lines from Zimbabwe and *ladybird* sequences from GenBank (accession number NT_033777.2). For other comments see Table S1.
