# Supplementary material for: Complex Interplay of Evolutionary Forces in the ladybird Homeobox Genes of Drosophila melanogaster
Source: PLoS One. 2011 Jul 22;6(7):e22613. doi: 10.1371/journal.pone.0022613 (PMC3142176; doi:10.1371/journal.pone.0022613)
Supplement: Table S4 — Recombination estimates ( ρ ). (DOC) [file pone.0022613.s007.doc]

**Table S4.** Recombination estimates (*ρ*)

|  | Full sequence | | | *lbe* | | | *lbl* | | |
| --- | --- | --- | --- | --- | --- | --- | --- | --- | --- |
|  | Per gene | Per site | *ρ*/θ | Per gene | Per site | *ρ*/θ | Per gene | Per site | *ρ*/θ |
| BAR | 53.707 | 0.0135 | 2.0719 | 9.018 | 0.0046 | 0.7834 | 49.098 | 0.0241 | 3.3477 |
| ER | 59.319 | 0.0148 | 2.5498 | 11.022 | 0.0056 | 1.1241 | 22.445 | 0.0109 | 1.6589 |
| VEN | 2.405 | 0.0006 | 0.1711 | 1.202 | 0.0006 | 0.1908 | 3.808 | 0.0019 | 0.4898 |
| Total | 60.922 | 0.0153 | 2.5932 | 11.623 | 0.0060 | 1.2196 | 33.267 | 0.0163 | 2.4375 |

Population recombination rate, *ρ*, is 4*N_e_r* (*N_e_* is effective population size and *r* is recombination rate / nucleotide site / generation) obtained by the method of McVean et al. [97]. All sites are included in the recombination analysis. Indels are excluded from the analyzed sequences. For other comments see Table S3.
