# Supplementary material for: Complex Interplay of Evolutionary Forces in the ladybird Homeobox Genes of Drosophila melanogaster
Source: PLoS One. 2011 Jul 22;6(7):e22613. doi: 10.1371/journal.pone.0022613 (PMC3142176; doi:10.1371/journal.pone.0022613)
Supplement: Table S5 — Gene conversion events in the lbe and lbl genes of D. melanogaster . (DOC) [file pone.0022613.s008.doc]

**Table S5.** Gene conversion events in the *lbe* and *lbl* genes of *D. melanogaster*

| Populations | *lbe* | | |  | *lbl* | | |
| --- | --- | --- | --- | --- | --- | --- | --- |
|  | N | *P* | L |  | N | *P* | L |
| BAR | 5 | 0.0113 | 1143 |  | 32 | 0.0006 | 691 |
| ER | 9 | 0.0136 | 1260 |  | 18 | 0.0002 | 882 |
| VEN | N. S. | | |  | 4 | 0.0364 | 536 |

The table gives number and significance of fragments involved in gene conversion events.

N, number of fragments. *P*-values are based on 10,000 permutations.

*L*, average length of the significant fragments. For other comments see Table S3.
