# Supplementary material for: Complex Interplay of Evolutionary Forces in the ladybird Homeobox Genes of Drosophila melanogaster
Source: PLoS One. 2011 Jul 22;6(7):e22613. doi: 10.1371/journal.pone.0022613 (PMC3142176; doi:10.1371/journal.pone.0022613)
Supplement: Table S6 — Linkage disequilibrium between functional regions of the lbe and lbl genes. (DOC) [file pone.0022613.s009.doc]

**Table S6.** Linkage disequilibrium between functional regions in the *lbe* and *lbl* genes of

*D. melanogaster*

|  | *lbl* intron I | *lbl* exon II | *lbl* intron II | *lbl* exon III | *lbl* 3’-fl. | Total |
| --- | --- | --- | --- | --- | --- | --- |
| *lbe* intron I | 11 | 1 | 42 | 14 | 12 | 80 |
| *lbe* exon II | 5 | 2 | 54 | 16 | 4 | 81 |
| *lbe* 3’-fl. | 23 | 6 | 84 | 28 | 27 | 168 |
| Total | 39 | 9 | 180 | 58 | 43 | 329 |

The table gives number of significant intergenic associations assessed by the Fisher exact test.
