# Supplementary material for: Complex Interplay of Evolutionary Forces in the ladybird Homeobox Genes of Drosophila melanogaster
Source: PLoS One. 2011 Jul 22;6(7):e22613. doi: 10.1371/journal.pone.0022613 (PMC3142176; doi:10.1371/journal.pone.0022613)
Supplement: Table S7 — Kelly's (Kelly 1997) and Wall’s (Wall 1999) tests of neutrality for the lbe and lbl gene regions. (DOC) [file pone.0022613.s010.doc]

**Table S7.** Kelly’s (Kelly 1997) and Wall’s (Wall 1999) tests of neutrality for the *lbe* and *lbl* gene regions of *D. melanogaster*

|  | *lbe* | | | |  | *lbl* | | | |  | *lbe* & *lbl* |
| --- | --- | --- | --- | --- | --- | --- | --- | --- | --- | --- | --- |
|  | Intron I | Coding | 3’-fl. | Full length |  | Intron I | Coding | 3’-fl. | Full length |  |  |
| Barcelona |  |  |  |  |  |  |  |  |  |  |  |
| *Z_nS_* | 0.1404 | 0.6352** | 0.3642 | 0.2684** |  | 0.2525 | 0.5428 | 0.6681*** | 0.2181** |  | 0.1661** |
| *B* | 0 | 0 | 0.1538 | 0.0741 |  | 0.2857 | 0.3333 | 0.4444** | 0.3235**** |  | 0.2097** |
| *Q* | 0 | 0 | 0.2143 | 0.1071 |  | 0.3750 | 0.5000 | 0.6000** | 0.5143**** |  | 0.3333** |
| El Rio |  |  |  |  |  |  |  |  |  |  |  |
| *Z_nS_* | 0.2947 | 0.8530*** | 0.3501* | 0.3652***** |  | 0.2558 | 0.5593* | 0.6920**** | 0.1867** |  | 0.1471**** |
| *B* | 0 | 0.2500 | 0.1538 | 0.1667 |  | 0.3636* | 0.2500 | 0.5556*** | 0.3488**** |  | 0.2794**** |
| *Q* | 0 | 0.4000 | 0.2143 | 0.2400 |  | 0.5000* | 0.4000 | 0.6000*** | 0.4545*** |  | 0.3768**** |
| Venezuela |  |  |  |  |  |  |  |  |  |  |  |
| *Z_nS_* | 0.3863 | 1.000*** | 0.5406*** | 0.5576***** |  | 0.6167*** | n. a. | 0.4297* | 0.3977**** |  | 0.3513***** |
| *B* | 0.5000** | 1.000** | 0.7273**** | 0.7000***** |  | 0.5714*** | n. a. | 0.2000 | 0.3182*** |  | 0.4884***** |
| *Q* | 0.8000** | 1.000** | 0.9167***** | 0.8095***** |  | 0.6250** | n. a. | 0.3636 | 0.4348*** |  | 0.5909***** |

**Table S7 (continued).**

|  | *lbe* | | | |  | *lbl* | | | |  | *lbe* & *lbl* |
| --- | --- | --- | --- | --- | --- | --- | --- | --- | --- | --- | --- |
|  | Intron I | Coding | 3’-fl. | Full length |  | Intron I | Coding | 3’-fl. | Full length |  |  |
| Total |  |  |  |  |  |  |  |  |  |  |  |
| *Z_nS_* | 0.1424 | 0.4344** | 0.3154** | 0.2343***** |  | 0.1705 | 0.4812* | 0.3989*** | 0.1361** |  | 0.1079* |
| *B* | 0.1000 | 0.1667 | 0.1429 | 0.1250 |  | 0.2500 | 0 | 0.0909 | 0.1277 |  | 0.1250* |
| *Q* | 0.1818 | 0.2857 | 0.2000 | 0.1818 |  | 0.3846 | 0 | 0.1667 | 0.2083 |  | 0.1975* |

Expected values of *Z_nS_* (Kelly 1997), *B*, and *Q* (Wall 1999) are obtained by coalescent simulations with recombination rate equal to 0.0153. Indels and singleton mutations are excluded from the analysis. **P* < 0.05; ***P* < 0.025; ****P* < 0.01; *****P* < 0.001; ******P* < 0.0001. n. a.: not applicable.
