# Supplementary material for: Complex Interplay of Evolutionary Forces in the ladybird Homeobox Genes of Drosophila melanogaster
Source: PLoS One. 2011 Jul 22;6(7):e22613. doi: 10.1371/journal.pone.0022613 (PMC3142176; doi:10.1371/journal.pone.0022613)
Supplement: Table S8 — Statistical significance of the binding site cluster for the lbe and lbl genes. (DOC) [file pone.0022613.s011.doc]

**Table S8.** Statistical significance of the binding site cluster for the *lbe* and *lbl* genes of *D. melanogaster*

|  | *lbe* |  | *lbl* |
| --- | --- | --- | --- |
|  | Score |  | Score |
| Adf | 4.47 |  | 4.00 |
| Aef | 0.96 |  | 2.32 |
| Ap | 3.98 |  | 2.00 |
| Br-Z1 | 1.50 |  | 2.86 |
| Br-Z2 | 1.85 |  | 3.82 |
| Br-Z3 | 3.64 |  | 4.58 |
| Br-Z4 | 2.25 |  | 3.07 |
| Dip3 | 4.50 |  | 4.44 |
| Dref | 1.33 |  | 2.50 |
| Hr46 | 4.25 |  | 2.25 |
| Kr | 2.87 |  | 1.97 |
| Pros | 1.88 |  | 3.01 |
| Su | 3.68 |  | 3.57 |
| bab1 | 4.01 |  | 4.67 |
| bcd | 1.46 |  | 2.97 |
| bin | 1.89 |  | 3.99 |
| brk | 4.21 |  | 2.37 |
| byn | 2.08 |  | 3.04 |

**Table 8 (continued).**

|  | *lbe* |  | *lbl* |
| --- | --- | --- | --- |
|  | Score |  | Score |
| cad | 1.20 |  | 1.69 |
| cfII | 3.42 |  | 4.38 |
| croc | 2.93 |  | 2.91 |
| d-mtTFA | 2.98 |  | 2.37 |
| dif | 1.67 |  | 0.38 |
| dl-A | 2.33 |  | 3.19 |
| dl-B | 2.13 |  | 2.31 |
| dri | 3.45 |  | 2.38 |
| en | 2.98 |  | 2.94 |
| esg | 4.14 |  | 3.99 |
| eve | 4.23 |  | 2.48 |
| gcm | 3.81 |  | 4.92 |
| hb | 2.82 |  | 3.63 |
| ovo | 4.33 |  | 2.46 |
| pan | 4.20 |  | 3.25 |
| pho | 4.65 |  | 2.22 |
| rel | 4.15 |  | 1.82 |
| sd | 3.13 |  | 3.39 |
| shn-ZFP1 | 2.00 |  | No clusters |

**Table 8 (continued).**

|  | *lbe* |  | *lbl* |
| --- | --- | --- | --- |
|  | Score |  | Score |
| shn-ZFP2 | 3.07 |  | 2.56 |
| slbo | 2.55 |  | 2.07 |
| srp | 1.97 |  | 2.08 |
| tin | 3.11 |  | 3.79 |
| tll | 3.33 |  | 5.15 |
| usp | 2.15 |  | 3.27 |
| zen | 4.23 |  | 2.48 |

Data on the binding site motifs are from C. Bergman (FlyReg database curated motifs; www.flyreg.org).
