# Supplementary material for: Complex Interplay of Evolutionary Forces in the ladybird Homeobox Genes of Drosophila melanogaster
Source: PLoS One. 2011 Jul 22;6(7):e22613. doi: 10.1371/journal.pone.0022613 (PMC3142176; doi:10.1371/journal.pone.0022613)
Supplement: Text S1 — Nucleotide polymorphism. (DOC) [file pone.0022613.s012.doc]

**Supporting information online, Text S1.**

Nucleotide polymorphism

There was no noticeable difference in the ratio of divergence to polymorphism *K*/π for the noncoding regions of the *lb* genes, but in the coding regions the synonymous *K*/π ratio was 2.0 – 2.7 times higher for *lbl* than for *lbe* (Tables 1 and 2). The difference was mostly due to variation in *lbl* exon II, where synonymous *K*/π = 18.89 (*D. melanogaster* – *D. simulans*) and 26.35 (*D. melanogaster* – *D. sechellia*). These differences suggest that the pressure to conserve synonymous sites of the coding region is higher for *lbl* than for *lbe*. For *lbl*, the strongest purifying selection was on exon II, which encompassed most of the homeodomain. Concordantly, the silent divergence in *lbl* was ~ 4 times higher in exon II than in exon III (see *K_mel-sim_* and *K_mel-sec_* in Table 2) despite similar synonymous variability in these coding regions. In the intron and 3’-flanking regions of *lb*, divergence between *D. melanogaster* and *D. simulans* or *D. sechellia* was less than half that between *D. melanogaster* and *D. yakuba*. In the coding region divergence between species was less pronounced, especially for synonymous sites of *lbe* and nonsynonymous sites of *lbl* (Tables 1 and 2).

Total nucleotide diversity for both genes was highest in Barcelona (π = 0.0070), lower in North America (π = 0.0058) and lowest in Venezuela (π = 0.0041) (Tables S1, S2, and S3). The same tendency was detected for the *esterase* gene region and explained as a bottleneck effect [1,2]. The most pronounced difference between populations was in the coding region of both *lbe* and *lbl* genes, which was significantly less polymorphic in South America than in Europe or North America (Tables S1, S2, and S3).

Total and noncoding divergence between *D. melanogaster* and *D. simulans* or *D. sechellia* was similar for *lbe* and *lbl* (Tables 1 and 2). Synonymous divergence was twice as high for *lbe* than for *lbl*. The pattern of divergence between *D. melanogaster* and *D. yakuba* was different: total and noncoding divergence was higher for *lbl*, but synonymous divergence was higher for *lbe* (Tables 1 and 2).

**References**

1. Balakirev ES, Ayala FJ (2003) Nucleotide variation of the *Est-6* gene region in natural populations of *Drosophila melanogaster*. Genetics 165:1901-1914.

2. Balakirev ES, Ayala FJ (2004) The *β-esterase* gene cluster of *Drosophila melanogaster*: Is ψ*Est-6* a pseudogene, a functional gene, or both? Genetica 121:165-179.
