# Supplementary material for: Complex Interplay of Evolutionary Forces in the ladybird Homeobox Genes of Drosophila melanogaster
Source: PLoS One. 2011 Jul 22;6(7):e22613. doi: 10.1371/journal.pone.0022613 (PMC3142176; doi:10.1371/journal.pone.0022613)
Supplement: Text S2 — Haplotype structure. (DOC) [file pone.0022613.s013.doc]

**Supporting information online, Text S2.**

Haplotype Structure

The presence of two or more highly diverged haplotypes has been interpreted as a result of positive selection in *D. melanogaster* [e.g., 1,2] or non-adaptive processes involving admixture between differentiated populations [3] or suppression of recombination caused by transposable elements [4]. Our data for seven *D. melanogaster* genes on the third chromosome, *Sod*, *Est-6*, ψ*Est-6, tinman*, *bagpipe*, *lbe*, and *lbl* [5-9; and present data] support the admixture hypothesis (possible historical admixture with genetically diverged population(s)). In addition to admixture, we suggest that local recombination plays an important role in shaping haplotype pattern because the distribution of diverged haplotypes across the genes studied is specific and not congruent. Our data do not exclude the transposon hypothesis. However there was only a single example of transposon insertion (*mdg-3* retrotransposon) detected for the ψ*Est-6* gene in a single *D. melanogaster* strain (out of 80 studied) [10].

**References**

1. Hudson RR, Bailey K, Skarecky D, Kwiatowski J, Ayala FJ (1994) Evidence for positive selection in the superoxide dismutase *Sod* region of *Drosophila melanogaster*. Genetics 136:1329-1340.

2. Bénassi V, Depaulis F, Meghlaoui GK, Veuille M (1999) Partial sweeping of variation at the *Fbp2* locus in a West African population of *Drosophila melanogaster*. Mol Biol Evol 16:347-353.

3. Teeter K, Naeemuddin M, Gasperini R, Zimmerman E, White KP, et al. (2000) Haplotype dimorphism in a SNP collection from *Drosophila melanogaster*. J Exp Zool 288:63-75.

4. Macpherson JM, Gonz**ález J, Witten DM, Davis JC, Rosenberg NA, et al. (2008)** Nonadaptive explanations for signatures of partial selective sweeps in Drosophila. Mol Biol Evol 25:1025-1042.

5. Balakirev ES, Ayala FJ (2003) Nucleotide variation of the *Est-6* gene region in natural populations of *Drosophila melanogaster*. Genetics 165:1901-1914.

6. Balakirev ES, Ayala FJ (2004) The *β-esterase* gene cluster of *Drosophila melanogaster*: Is ψ*Est-6* a pseudogene, a functional gene, or both? Genetica 121:165-179.

7. Balakirev ES, Balakirev EI, Rodriguez-Trelles F, Ayala FJ (1999) Molecular evolution of two linked genes, *Est-6* and *Sod*, in *Drosophila melanogaster*. Genetics 153:1357-1369.

8. Balakirev ES, Balakirev EI, Ayala FJ (2002) Molecular evolution of the *Est-6* gene in *Drosophila melanogaster*: Contrasting patterns of DNA variability in adjacent functional regions. Gene 288:167-177.

9. Balakirev ES, Ayala FJ (2004) Nucleotide variation in the *tinman* and *bagpipe* homeobox genes of *Drosophila melanogaster*. Genetics 166:1845-1856.

10. Balakirev ES, Chechetkin VR, Lobzin VV, Ayala FJ (2003) DNA polymorphism in the *β-esterase* gene cluster of *Drosophila melanogaster*. Genetics 164:533-544.
