# Supplementary material for: Complex Interplay of Evolutionary Forces in the ladybird Homeobox Genes of Drosophila melanogaster
Source: PLoS One. 2011 Jul 22;6(7):e22613. doi: 10.1371/journal.pone.0022613 (PMC3142176; doi:10.1371/journal.pone.0022613)
Supplement: Text S3 — Linkage disequilibrium. (DOC) [file pone.0022613.s014.doc]

**Supporting information online, Text S3.**

Linkage Disequilibrium

Linkage disequilibrium (LD) was evaluated using Fisher’s exact test for all pairs between polymorphic sites (excluding singletons). Among 3240 pairwise comparisons for the *lb* region, 32.84 % were significant with 7.8 % significant after the stringent Bonferroni correction (Fig. S3). There was strong LD within *lbe*: 27.65 % (146 out of 528) pairwise comparisons were significant after Bonferroni correction. Within *lbe* intron I and exon II, density of significant associations was similar, but within the 3’-flanking region it was almost twice as high. Within *lbl*, 12.41 % (140 out of 1128) pairwise comparisons showed statistically significant LD (with Bonferroni correction). Distribution of significant associations within *lbl* was not homogeneous: intron I and the 3’-flanking region had similar density of significant associations; exonic region – minimum number, and intron II – maximum (Fig. S3). Between the *lbe* and *lbl* genes 10.2 % (329 out of 3240) tests were significant (shaded in Fig. S3; see also Table S6), which amount to 30.9 % of total number of significant associations in *lbe* and *lbl* (without Bonferroni correction). Proportion of significant associations was higher for *lbe* (0.33) than for *lbl* (0.22), which corresponds to the recombination rate differences between the two genes (Table S4). In general, level of LD for *lb* genes was similar to that in other members of the 93DE gene cluster (*tin* and *bap* genes) [1]. Level of between-genes associations was more pronounced for *lbe* and *lbl* (10.15 % significant associations) than for *tin* and *bap* (5.22 % significant associations).

**References**

1. Balakirev ES, Ayala FJ (2004) Nucleotide variation in the *tinman* and *bagpipe* homeobox genes of *Drosophila melanogaster*. Genetics 166:1845-1856.
