# Supplementary material for: Complex Interplay of Evolutionary Forces in the ladybird Homeobox Genes of Drosophila melanogaster
Source: PLoS One. 2011 Jul 22;6(7):e22613. doi: 10.1371/journal.pone.0022613 (PMC3142176; doi:10.1371/journal.pone.0022613)
Supplement: Text S4 — Neutrality and maximum likelihood (ML) analysis of selective pressures. (DOC) [file pone.0022613.s015.doc]

**Supporting information online, Text S4.**

Tests of Neutrality and Maximum Likelihood (ML) Analysis of Selective Pressures

To verify the robustness of ML estimation of mutation fitness parameters, we fitted the model to each dataset in two ways: (1) simultaneously optimized model parameters and branch lengths, and (2) optimized branch lengths under one-ratio model M0, then fixed branch lengths to ML estimates and used FMutSel to optimize model parameters. ML estimates of model parameters were always very close for the two routines (typically differed in the second decimal). We investigated the estimated distributions of population scaled selection coefficients *S_IJ_* = *F_I_* – *F_J_* for mutations between codons *I* and *J*, with *F_I_* = 2*Nf_I_* being a population scaled fitness of codon *I*. When the full *lbe* sample was analyzed, 27% of all possible 526 mutations (all possible types of mutations between codons allowed by the model of [1]) were estimated to have large fitness effects (|*S_IJ_* |>2), but the estimate sharply increased to 100% when only *D. melanogaster* samples (70 strains or 60 non-recombinants) were analyzed. Moreover, all mutations seen within *lbe* of *D. melanogaster* samples showed detrimental fitness effects. Notice that the fitness of each mutation in the FMutSel model was independent of the location of the site, as it was assumed constant across the sequence. There was no reason why this should be the case: a codon preferred in most sites in the sequence may be selected against at a handful of other sites, e.g., to reduce speed of translation in order for the protein to obtain the correct folding.

In *lbl*, proportion of mutations with large fitness effects was 91% for the full sample, which increased to 98% when only *D. melanogaster* strains were analyzed. For mutations with a negative effect, mean *S_IJ_* was –6.7, for *D. melanogaster*, lower (in absolute value) than the –13.1 estimate for *lbe*. 0.5% mutations in *lbl* have strong positive fitness effect, with mean *S_IJ_* for all mutations with positive effect 2.0.

Estimates of selective coefficients for mutations in *lb* genes are in contrast with estimates for *Est-6* and ψ*Est-6* (*tin* and *bap* are not analyzed due to small sample size for this parameter-rich model). 20% of the mutations had large fitness effects (|*S_IJ_* |>2) in *Est-6*, with only 3% of such mutations in ψ*Est-6* (which is relevant to the debate on the functional redundancy of this gene; [2,3]). Mean selection coefficients for advantageous mutations were 0.8 in *Est-6* and 0.6 in ψ*Est-6*, while for deleterious mutations they were –1.5 in *Est-6* and –1.0 in ψ*Est-6*. Absolute values of these estimates were < 2, which implies that their selective effect was likely very mild. In contrast, the majority of mutations in *lb* genes had strong selective effects.

**References**

1. Yang Z, Nielsen R (2008) Mutation-selection models of codon substitution and their use to estimate selective strengths on codon usage. Mol Biol Evol 25:568-579.

2. Balakirev ES, Ayala FJ (1996) Is esterase-P encoded by a cryptic pseudogene in *Drosophila melanogaster*? Genetics 144:1511-1518.

3. Balakirev ES, Ayala FJ (2004) The *β-esterase* gene cluster of *Drosophila melanogaster*: Is ψ*Est-6* a pseudogene, a functional gene, or both? Genetica 121:165-179.
